# Dynamic Simulation and Metabolome Analysis of Long-Term Erythrocyte Storage in Adenine–Guanosine Solution

**DOI:** 10.1371/journal.pone.0071060

**Published:** 2013-08-16

**Authors:** Taiko Nishino, Ayako Yachie-Kinoshita, Akiyoshi Hirayama, Tomoyoshi Soga, Makoto Suematsu, Masaru Tomita

**Affiliations:** 1 Institute for Advanced Biosciences, Keio University, Tsuruoka, Yamagata, Japan; 2 Systems Biology Program, Graduate School of Media and Governance, Keio University, Fujisawa, Kanagawa, Japan; 3 Institute of Biomaterials and Biomedical Engineering, University of Toronto, Toronto, Onatrio, Canada; 4 Department of Biochemistry, School of Medicine, Keio University, Shinjuku, Tokyo, Japan; 5 Faculty of Environment and Information Studies, Keio University, Fujisawa, Kanagawa, Japan; University of Catania, Italy

## Abstract

Although intraerythrocytic ATP and 2,3-bisphophoglycerate (2,3-BPG) are known as direct indicators of the viability of preserved red blood cells and the efficiency of post-transfusion oxygen delivery, no current blood storage method in practical use has succeeded in maintaining both these metabolites at high levels for long periods. In this study, we constructed a mathematical kinetic model of comprehensive metabolism in red blood cells stored in a recently developed blood storage solution containing adenine and guanosine, which can maintain both ATP and 2,3-BPG. The predicted dynamics of metabolic intermediates in glycolysis, the pentose phosphate pathway, and purine salvage pathway were consistent with time-series metabolome data measured with capillary electrophoresis time-of-flight mass spectrometry over 5 weeks of storage. From the analysis of the simulation model, the metabolic roles and fates of the 2 major additives were illustrated: (1) adenine could enlarge the adenylate pool, which maintains constant ATP levels throughout the storage period and leads to production of metabolic waste, including hypoxanthine; (2) adenine also induces the consumption of ribose phosphates, which results in 2,3-BPG reduction, while (3) guanosine is converted to ribose phosphates, which can boost the activity of upper glycolysis and result in the efficient production of ATP and 2,3-BPG. This is the first attempt to clarify the underlying metabolic mechanism for maintaining levels of both ATP and 2,3-BPG in stored red blood cells with *in silico* analysis, as well as to analyze the trade-off and the interlock phenomena between the benefits and possible side effects of the storage-solution additives.

## Introduction

In the last 3 decades, various additive solutions for blood storage have been developed to prevent storage lesions, including metabolic or physiologic changes. The principal indicators of metabolic deterioration are the decrease in adenosine-5′-triphosphate (ATP) and 2,3-bisphosphoglycerate (2,3-BPG) levels. ATP is known as a predictor of the viability of red blood cells (RBCs) after transfusion [1]. The loss of 2,3-BPG results in changes in hemoglobin oxygen affinity, which leads to the loss of oxygen delivery to tissues [2], [3]. Moreover, irreversible change in cell shape and loss of membrane plasticity are strongly associated with ATP depletion during storage [4]. Under these circumstances, efforts to improve RBC storage methods have focused on optimizing energy-producing ATP and 2,3-BPG [4]. However, current additive solutions do not maintain constant levels of ATP and 2,3-BPG in RBCs, leading to the rapid decrease in 2,3-BPG and a 30–50% decrease in ATP content over a storage period of 5–6 weeks [5]. In addition, the fate of additives in stored blood and behaviors of metabolic intermediates have received little attention, despite the evaluation of metabolic byproducts like inosine and hypoxanthine (HX) during cold-storage, which are rapidly converted to toxic uric acid in the circulation [6]. A mechanism of increase of the metabolic byproducts should be considered by observing the metabolic dynamics in a comprehensive manner. For example, adenine (ADE) is currently included in all commonly used RBC additive solutions due to its beneficial effect on ATP maintenance as a purine source; however, it is also known to break down into other metabolites like inosine and HX, which can be toxic owing to the increased potential for uric acid generation or oxidative stress. An excess increase in plasma uric acid in blood transfusion causes liver function disorder, and can be a risk factor for hyperuricemia.

Detailed mathematical models are useful to analyze complex metabolic regulations in functional organs, tissues, and cells [7], [8]. In a previous study, we developed a large-scale precise kinetic model for predicting the whole metabolic behavior of cold-stored RBCs with the commonly used additive solution in Japan [9]. From the *in silico* analysis, we suggested that the activation of phosphofructokinase (PFK) would lead to the maintenance of ATP and 2,3-BPG levels during RBC storage [9]. This prediction was confirmed by the experimental study of a novel additive solution phosphate-adenine-glucose-guanosine-gluconate-mannitol (PAGGGM) [10]. PAGGGM includes glucose, ADE, and guanosine (GUO) as energy substrates, and it is superior to conventional storage solutions for maintaining both ATP and 2,3-BPG levels over 5 weeks [5]. Burger and his colleagues observed the time-related changes of glycolytic intermediates in PAGGGM-stored RBCs and first provided the experimental evidence that PFK activity is likely to be enhanced in PAGGGM relative to conventional additive solutions [10]. A comprehensive simulation model can trace not only the time-related changes in metabolites' levels, but also those in enzymatic activities, which are difficult to observe in experimental procedure under blood storage condition, and helps us to understand the underlying regulatory mechanism of the associated maintenance of ATP and 2,3-BPG.

In this study, we predicted the metabolic dynamics of PAGGGM-stored RBCs with the large-scale *in silico* model in order to clarify the underlying mechanism of how 2 functional metabolites, namely, ATP and 2,3-BPG, can be maintained over a long storage period. We also determined the comprehensive roles of solution additives in the metabolic dynamics of cold-storage RBCs. Through the simulation analysis, we predicted the mechanism of PFK activation, as well as the fate of 2 major additives, ADE and GUO, for RBCs in cold storage conditions in PAGGGM solution. A model of PAGGGM-stored RBCs was developed based on a curated RBC metabolic model developed by Kinoshita et al. [8] by changing environmental components and pH- or temperature-related enzymatic activities. The model prediction of overall metabolic changes over storage time was validated by experimental measurements of metabolic intermediates by capillary electrophoresis time-of-flight mass spectrometry (CE-TOFMS) analysis. The metabolic behaviors of PAGGGM-stored RBC predicted by the model were in qualitatively agreement with that measured by CE-TOFMS analysis. From the simulation analysis, we concluded that ADE is useful for the maintenance of the adenylate pool, which can increase ATP levels, but causes an accumulation of metabolic waste like HX. Moreover, ADE induces the consumption of ribose phosphates, which leads to 2,3-BPG reduction. In contrast, GUO is used as a source of ribose phosphates in the non-oxidative pentose phosphate pathway (non-ox PPP) without increasing purine metabolites. Increasing concentrations of ribose phosphates result in an accumulation of upper glycolytic intermediates, leading to PFK activation and an associated increase in both ATP and 2,3-BPG. This indicates that GUO can compensate for the depletion of 2,3-BPG induced by ADE. Further analysis suggested that the fate of GUO itself is also controlled by ADE concentration. The trade-offs and coordinating mechanism between ADE and GUO, which provide metabolic benefits and disadvantages under RBC storage conditions, are discussed herein.

## Materials and Methods

### Ethics Statement

All procedures for blood sample collection were approved by the research ethics committee of Keio University School of Medicine, and all participants gave written informed consent.

### Blood collection and preservation

Whole blood samples were collected from healthy volunteers by using vacuum blood collection tubes with sodium heparin (Venoject II; Terumo Corp., Tokyo, Japan). Blood samples were washed and suspended in citrate phosphate dextrose (CPD) solution (26.3 g/L sodium citrate hydrate, 3.27 g/L citric acid hydrate, 23.2 g/L glucose, and 14.57 g/L sodium dihydrogen phosphate). The blood suspended in CPD was centrifuged at 3300×*g* for 10 min, and the plasma and buffy coat were removed. The buffy coat–free blood was suspended in PAGGGM solution (8.726 g/L sodium gluconate, 0.195 g/L ADE, 8.558 g/L glucose, 0.408 g/L GUO, 9.909 g/L mannitol, 1.248 g/L NaH_2_PO_4_·H_2_O, and 0.2855 g/L NaH_2_PO_4_·12H_2_O) [5], resulting in a hematocrit of 60%. Finally, RBCs suspended in PAGGGM solution were divided into 1-mL plastic tubes and stored at 4°C for 0–35 days.

### Measurement of intracellular pH

Intracellular pH was measured as described previously [11]. Briefly, 900 µL of PAGGGM-RBC was centrifuged at 3300×*g* for 5 min. The supernatant solution was removed and 100 µL erythrocytes hemolyzed by freezing in liquid nitrogen. Milli-Q water (100 µL) was added, and the pH of the lysate was measured by using a portable blood gas analyzer i-STAT 300F (Fuso Pharmaceutical Industries, Osaka, Japan) at days 0–14 or by using a compact pH meter (HORIBA B-211) at days 21–35.

### Preparation of erythrocytes for capillary electrophoresis time-of-flight mass spectrometry analysis

To isolate erythrocytes, PAGGGM-RBC was centrifuged at 3300×*g* and 4°C for 5 min, and the supernatant solution was removed. Next, 0.2 mL of the sample erythrocyte pellet was treated with 1.8 mL cold methanol containing 20 μM each of methionine sulfone and d-camphor-10-sulfonic acid (CSA) as the internal standard. Chloroform (2 mL) and Milli-Q water (0.8 mL) were added, and the mixture was thoroughly mixed and then centrifuged at 5800×*g* for 5 min at 4°C. Next, the upper aqueous layer was filtered through a centrifugal filter (Millipore, 5-kDa cut-off filter) to remove proteins. The filtrate was concentrated centrifugally and then dissolved in 50 µL of Milli-Q water containing reference compounds (200 µM each of 3-aminopyrrolidine and trimesate) immediately prior to CE-TOFMS analysis [12].

### Instrumentation

All CE-TOFMS experiments were performed using an Agilent CE Capillary Electrophoresis System equipped with a G3250 AA LC/MSD TOF system, 1100 isocratic HPLC pump, G1603A CE-MS adapter kit, and G1607A CE-electrospray ionization (ESI)-MS sprayer kit (Agilent Technologies, Waldbronn, Germany). System control and data acquisition were performed using Agilent G2201AA ChemStation software for CE and Analyst QS for Agilent TOFMS.

### CE-TOFMS conditions for cationic/anionic metabolites and nucleotides analysis

Separations of cationic metabolites were carried out in a fused-silica capillary (50 μm inner diameter×103 cm total length) filled with 1 M formic acid as the reference electrolyte [13]. For anionic metabolites and nucleotides separations, a cationic polymer–coated COSMO(+) capillary (50 μm inner diameter×106 cm total length; Nakalai Tesque, Kyoto, Japan) was used. A 50 mM ammonium acetate solution (pH 8.5) was used as the reference electrolyte [14]. Other analytical conditions for ionic metabolites and nucleotides are the same as described in our recent paper [9].

### CE-TOFMS data processing

The obtained raw data was integrated by in-house software via the following steps: noise-filtering, baseline-correction, migration time alignment, peak detection, and integration of each peak area from sliced electropherograms (width of each electropherogram, 0.02 m/z). Subsequently, the accurate m/z for each peak was calculated with Gaussian curve fitting on the m/z domain peak. All metabolites were identified by matching the m/z values and migration times with the standard compounds.

### PAGGGM-stored RBC model construction

**Figure 1:**
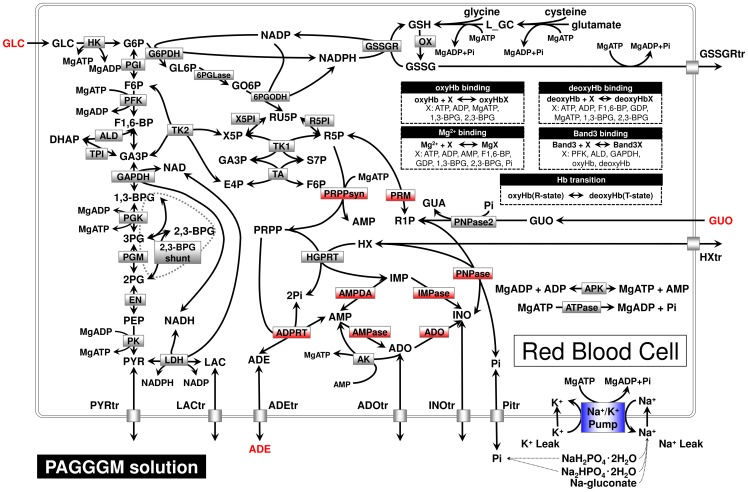
Pathway reactions in the mathematical model of PAGGGM-stored RBCs. Nodes indicate metabolites or ions, and edges indicate enzymatic reactions or transport processes, which were divided into 3 groups (*red, blue, gray boxes*) by the difference in sensitivity of enzymatic or reaction activity to temperature or pH as described in our previous study [9]. Abbreviations used in this figure are given in Table 1.

We built a PAGGGM-stored RBC model based on a published model of human erythrocyte metabolism [8]. In this study, all simulation experiments were carried out on E-Cell System Environment Version 3. In E-Cell 3 simulation platform, variable step-size ODE solver is implemented so that we can perform fast and accurate calculation [15]. The full set of ODEs, parameter list, and initial conditions of PAGGGM-stored RBC model are supplied in Text S1. The model written in SBML (XML) format is provided in Model S1. The basal model includes the major metabolic pathways such as glycolysis, pentose phosphate pathway, purine salvage pathway, membrane transport systems for intermediates, ion leak processes, ATP-dependent Na^+^/K^+^ pump process, and binding reactions between hemoglobin and metabolites. Figure 1 shows metabolic pathways included in the PAGGGM-stored RBC model. The PAGGGM solution comprised glucose, ADE and GUO as the energy substrates, Na_2_HPO_4_ and NaH_2_PO_4_ as the source of phosphate and as the pH buffer, and mannitol and sodium gluconate as osmoregulatory substrates.

**Table 1:** Enzymatic reactions included in the model a.

Enzyme/Process	Abbreviation	Substrates ^d^		Products ^e^	Effectors ^f^
Hexokinase	HK	GLC + MgATP	→	G6P + MgADP	2,3-BPG, GDP, GSH, pH
Phosphoglucoisomerase	PGI	G6P	↔	F6P	
Phosphofructokinase	PFK	F6P + MgATP	→	F1,6-BP + MgADP	fATP, Mg, 2,3-BPG, fAMP, Pi, GDP, pH
Aldolase	ALD	F1,6-BP	↔	DHAP + GA3P	2,3-BPG, Mg2,3-BPG
Triose phosphate isomerase	TPI	DHAP	↔	GA3P	
Glyceraldehyde phosphate dehydrogenase	GAPDH	GA3P + Pi + NAD	↔	1,3-BPG + NADH	pH
2,3-Bisphosphoglycerate shunt	BPGSP	1,3-BPG	→	2,3-BPG + 3PG + Pi	pH
Phosphoglycerate kinase	PGK	1,3-BPG + MgADP	↔	3PG + Pi	
Phosphoglyceromutase	PGM	3PG	↔	2PG	
Enolase	EN	2PG	↔	PEP	Mg
Pyruvate kinase	PK	PEP + MgADP	↔	PYR + MgATP	ATP, F1,6-BP, GDP, pH
Lactate dehydrogenase	LDH	PYR + NADH	→	LAC + NAD	pH
Lactate dehydrogenase (NADPH)	LDHP	PYR + NADPH	→	LAC + NADP	
Glucose 6-phosphate dehydrogenase	G6PDH	G6P + NADP	→	GL6P + NAPDH	MgATP, 2,3-BPG
6-Phosphogluconolactonase	6PGLase	GL6P	→	GO6P	
6-Phosphogluconate dehydrogenase	6PGODH	GO6P + NADP	↔	RU5P + NADPH + CO2	
Transaldolase	TA	S7P + GA3P	→	E4P + F6P	
Transketolase1	TK1	X5P + R5P	→	GA3P + S7P	
Transketolase2	TK2	X5P + E4P	→	GA3P + F6P	
Ribose 5-phosphate isomerase	R5PI	RU5P	→	R5P	
Xylulose 5-phosphate isomerase	X5PI	RU5P	→	X5P	
Gamma-glutamyl cysteine synthetase	L_GCS	MgATP + glutamate + cysteine	↔	MgATP + L_GC + Pi	GSH
Glutathione synthetase	GSH_S	L_GC + glycine + MgATP	↔	GSH + MgATP + Pi	
Glutathione reductase	GSSGR	GSSG + NADPH	→	GSH + MgADP + Pi	
Adenosine kinase	AK	ADO + MgATP	→	MgADP + fAMP	
Hypoxanthine-guanine phosphoryl transferase	HGPRT	PRPP + HXi	→	IMP + 2Pi	
Adenosine deaminase^b^	ADA	ADO + MgATP	↔	INO	
Adenine phosphoribosyl transferase^b^	ADPRT	ADE + PRPP	→	fAMP + 2Pi	
Adenosine monophosphate deaminase^b^	AMPDA	fAMP	→	IMP	
Adenosine monophosphate phosphohydrolase^b^	AMPase	fAMP	↔	ADO + Pi	
Inosine monophosphatase^b^	IMPase	IMP	↔	INO + Pi	
Purine nucleoside phophorylase^b^	PNPase	INO + Pi	→	HXi + R1P	
Phosphoribomutase^b^	PRM	R1P	→	R5P	
Phosphoribosyl pyrophosphate synthetase^b^	PRPPsyn	R5P + MgATP	→	PRPP + fAMP + Mg	
Purine nucleoside phophorylase	PNPase2	GUO + Pi	→	GUA + R1P	
Adenylate kinase	APK	ADP + MgADP	→	AMP + MgATP	
Adenosine triphosphate phosphohydorolase	ATPase	MgATP	→	MgADP + Pi	
Glutathione turnover	OX	2GSH	→	GSSG	
Non-glycolytic NADH consumption process	OXNADH	NADH	→	NAD	
Adenine transport process	ADEtr	ADE	→	ADEe	
Adenosine transport process	ADOtr	ADO	→	ADOe	
Hypoxanthine transport process	HXtr	HXi	→	HXe	ADEe
Inosine transport process	INOtr	INO	→	INOe	
Lactate transport process	LACtr	LAC	→	LACe	
Pyruvate transport process	PYRtr	PYR	→	PYRe	
Inorganic phosphate transport process	Pitr	Pi	→	Pie	
GSSG transport process	GSSG transport	GSSG + MgATP	→	GSSGe + MgADP + Pi	
Leak of potassium	K_Leak	Ke	↔	Ki	
Leak of sodium	Na_Leak	Nae	→	Nai	
Sodium/potassium pump^c^	Na^+^/K^+^ pump	3Nai + 2Ke + MgATP	→	3Nae + 2Ki + MgADP + Pi	

a From [8].

b,c The enzyme activities in the model were divided into 3 groups: activities of 8 enzymes in purine salvage pathway (b), the activity of the Na^+^/K^+^ pump (c), and the remaining other enzymatic or binding activities [9].

d,e,f Abbreviations used in this table are as follows: GLC, glucose; G6P, glucose 6-phosphate; F6P, fructose 6-phosphate; F1,6-BP, fructose 1,6-bisphosphate; DHAP, dihydroxyacetone phosphate; GA3P, glyceraldehyde 3-phosphate; 1,3-BPG, 1,3-bisphosphoglycerate; 2,3-BPG, 2,3-bisphosphoglycerate, 3PG, 3-phosphoglycerate; 2PG, 2-phosphoglycerate; PEP, phosphoenolpyruvate; PYR, pyruvate; LAC, lactate; GL6P, gluconolactone 6-phosphate; GO6P, gluconate 6-phosphate; RU5P, ribulose 5-phosphate; X5P, xylulose 5-phosphate; E4P, erythrose 4-phosphate; S7P, sedoheptulose 7-phosphate; R5P, ribose 5-phosphate; PRPP, 5-phosphoribosyl 1-phosphate; ADE, adenine; IMP, inosine monophosphate; R1P, ribose 1-phosphate; INO, inosine; ADO, adenosine; HX, hypoxanthine; AMP, adenosine monophosphate; ADP, adenosine diphosphate; ATP, adenosine triphosphate; NADP, nicotinamide adenine dinucleotide phosphate; NADPH, nicotinamide adenine dinucleotide phosphate (reduced); NAD, nicotinamide adenine dinucleotide; NADH, nicotinamide adenine dinucleotide (reduced); Ki, potassium ion; Nai, Sodium ion; Pi, inorganic phosphate; L_GC, l-glutamyl cysteine; GSH, glutathione (reduced); GSSG, glutathione (oxidized).

In human erythrocytes, GUO is converted into guanine and ribose 1-phosphate (R1P) by purine nucleoside phosphorylase [16]. Thus, in the present study, we modeled a process of GUO consumption as follows:
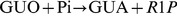
(1)

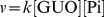
(2)


Phosphorylation rate constant (*k*  =  1e+8 s^−1^·M^−2^) is determined by manually fitting the GUO depletion curve as measured by CE-TOFMS experiments (Figure S1).

We modeled the tense–relax state transition of hemoglobin and inactivation of all chemical reactions induced by low temperatures in 4°C and additive solution during blood storage as per our previous study [9]. Insomuch as hemoglobin is known to be stable in the R-state at low temperature, we set all hemoglobin as in the R-state in PAGGGM-stored RBC model. At low temperatures, all chemical reactions such as enzymatic reactions, chemical-binding reactions, and active transport processes are significantly inactivated. According to our earlier study, all the chemical reactions were divided into 3 groups on the basis of their sensitivity to temperature and pH, such as Na^+^/K^+^ pump activity, purine salvage activities, and all other reaction activities [9]. The reaction activities of each group were set as 0.1%, 25.0%, and 3.0% of the values in the basal model (37°C), respectively, by retuning the previous model of cold-stored RBCs preserved in Mannitol-Adenine-Phosphate (MAP) solution in ref. [9]. The details of the parameter settings are described in Text S1. CE-TOFMS measurements showed that the peak intracellular concentrations of fructose-1,6-bisphosphate (F-1,6-BP), dihydroxyacetone phosphate (DHAP) and lactate (LAC) were significantly larger in PAGGGM solution compared with MAP solution (*p*-values <0.001) (Figure S2). These differences in the peak concentrations could be reproduced with the computational models by changing the initial concentrations of extracellular additives and the decline curve in intracellular pH, and by adding the simple first-order process of guanosine phosphorylation (Figure S2). The difference between the basal model (37°C), MAP-stored RBC model (4°C) and PAGGGM-stored RBC model (4°C) was showed in Table S1.

**Figure 2:**
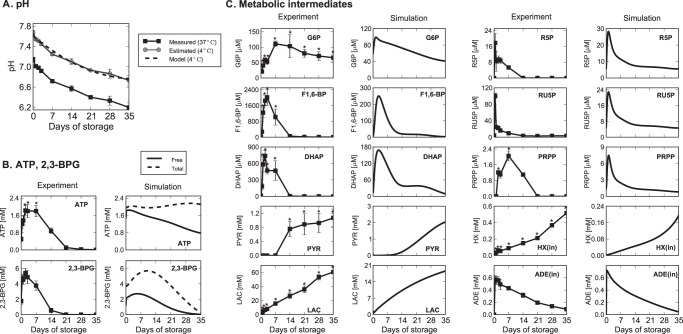
Measured and predicted time courses of pH and metabolic intermediates in PAGGGM-stored RBCs. *Panel A*: Time courses of intracellular pH at 37°C (measured values; ▪) and at 4°C (estimated values; •); fitted value used in the model (–). *Panel B*: ATP and 2,3-BPG were measured by CE-TOFMS (*left column*) and predicted by the mathematical model (*right column*) in which free-form (*solid black*) and total amount (*broken black*) of metabolites are shown separately. Since intracellular ATP and 2,3-bisphophoglycerate (2,3-BPG) are known to bind to hemoglobin and band 3 membrane protein [8], in our model, free-form metabolite represents its amount in plasma, and total amount of metabolite represents the sum of metabolites (free-form) in plasma and their binding to the proteins. *Panel C*: Measured or predicted time courses of intermediates in glycolysis (G6P, F1,6-BP, DHAP, PYR, LAC), non-oxidative pentose phosphate pathway (R5P, RU5P), and purine salvage pathway (PRPP, intracellular HX, and ADE), respectively. pH and capillary electrophoresis time-of-flight mass spectrometry (CE-TOFMS) data are expressed as means ± SD of 6 separate experiments. An asterisk indicates that a two-sided *p*-value is <0.01 versus the Day 0 values.



We also modeled the pH-degradation profile of PAGGGM-stored RBCs. The time courses of intracellular pH in PAGGGM-stored RBCs were measured at 37°C (Figure 2A). However, the physiological pH in cold-stored erythrocytes is known to be much higher than that measured at 37°C [17]. Therefore, we estimated the actual intracellular pH in PAGGGM-stored RBCs at 4°C by using the relationship of temperature and intracellular pH, i.e., Δ pH/°C  = −0.0161, proposed by Guppy et al. [17] (Figure 2A). The intracellular pH decline in PAGGGM-stored RBC was approximated as eq.(3) from the estimated pH time-series under 4°C.

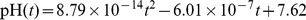
(3)Sensitivity analysis of all kinetic parameters in the PAGGGM-stored RBC model against time-courses of ATP and 2,3-BPG indicates that the model dynamics is enough robust to the kinetic parameters values (Figure S3).

### Statistical analyses

Paired Student's *t*-test was employed to analyse CE-TOF-MS time course data to show the differences from the value of Day0. A two-sided *p* value of <0.01 was considered statistically significant. Differences in mean metabolic concentrations between MAP-stored RBCs and PAGGGM-stored RBCs were examined using two-sample *t*-test.

## Results and Discussion

### Refinement of RBC metabolic model to represent dynamics of PAGGGM-stored RBC metabolism

The basal steady-state model assuming 37°C described in ref. [8] includes not only precise metabolic enzymatic reactions of comprehensive pathways but also other key chemical reactions producing metabolic changes, such as allosteric transition in hemoglobin and subsequent binding or disassociation between hemoglobin and ATP, 2,3-BPG/band3 membrane proteins/glycolytic enzymes. Each reaction in the basal model is described by detailed kinetic equations, where some enzymatic reactions in glycolysis, for instance, hexokinase, PFK, glyceraldehyde phosphate dehydrogenase, pyruvate kinase, lactate dehydrogenase, and reactions in 2,3-BPG shunt, are represented as pH-dependent kinetics. These features of the basal model are essential for the simulation of cold-stored RBCs, in which gradual but remarkable decline in pH, R-state stabilization of hemoglobin by low temperature, and non-uniform inactivation of chemical/enzymatic reactions occur at the same time. A PAGGGM-stored RBC model was developed by refinement of our previous model of cold-stored RBC metabolism in MAP-solution [9] to meet the difference in environmental or intracellular conditions. Specifically, we changed initial concentrations of extracellular additives, added the simple first-order process of GUO phosphorylation, and refitted the parameters of enzymatic activities (Figure S2) and the decline curve in intracellular pH. GUO phosphorylation rate constant was obtained from the fitting manually to the GUO depletion curve measured by CE-TOFMS (Figure S1). A parameter setting details is described inText S1. The sensitivity analysis of this rate constant indicated that GUO phosphorylation exerts influence on the dynamics of middle glycolytic intermediates and intracellular HX and ADE, but not on ATP, 2,3-BPG, upstream/downstream glycolytic intermediates (Figure S4).

### Measured and predicted time courses of metabolic intermediates in PAGGGM-stored RBCs

To validate the PAGGGM-stored RBC model, we measured time-course behaviors of intermediates in PAGGGM-stored RBCs during 35 days of cold storage. Figure 2B-C shows the results of CE-TOFMS measurements and model predictions of PAGGGM-stored RBCs.

Metabolome analysis provides us a comprehensive view of metabolic dynamics during blood storage. Unlike those of flowing RBCs, intracellular metabolites of PAGGGM-stored RBCs were not in a steady state but largely changed with storage time. The time-course changes of metabolite pools were clearly observed even in the cold temperature in which enzymatic activities are assumed to be very low; some metabolites were significantly accumulated, but others were exhausted under storage conditions. Under these circumstances, comprehensive kinetic model is useful to predict such non-linear dynamics of metabolic systems.

The predicted total amount of ATP was maintained at a high level during 35 days of storage, whereas the predicted total 2,3-BPG increased up to 1.5-fold during the first 1–2 weeks of storage and then gradually decreased (Figure 2B, *black broken curves*). These results agreed with those in the previous report by Burger *et* *al.* [10], in which ATP was kept at initial level throughout 35 days of storage, and 2,3-BPG was increased up to 1.3-fold of initial level during 21 days of storage and then rapidly decreased [10]. In contrast, our metabolome analysis showed that ATP and 2,3-BPG were almost depleted at days 21 and 14, respectively (Figure 2B). Large amounts of intracellular ATP and 2,3-BPG are known to bind to hemoglobin and band 3 membrane protein to modulate the oxygen absorbing capacity of hemoglobin, as well as to control glycolytic flux [8], and the time-series alterations measured by metabolome analysis resembled those for free-form ATP and 2,3-BPG as predicted by the model (Figure 2B, *black solid curves*). This result is consistent with a report that only free-form ATP and 2,3-BPG would be quantified in CE-TOFMS measurements, because hemoglobin-binding ATP and 2,3-BPG were lost during the protein denaturation step in the preparation of CE-TOFMS analysis [8].

The results of the CE-TOFMS analysis and model prediction clearly indicated that metabolic intermediates can be decomposed into certain metabolic pools according to their dynamics. The glycolytic intermediates were classified into 3 groups based on their time-course patterns: upper (glucose-6-phosphate; G6P, and fructose-6-phosphate; F6P), middle (F1,6-BP and DHAP), and lower (pyruvate; PYR, and LAC) stream of glycolysis (Figure 2C). According to both predictions and metabolome measurements, upper glycolytic intermediates increased within the first week of storage and then started to decrease moderately. Middle-glycolytic intermediates were significantly elevated during 2–3 days of storage (*p*-values <0.001 versus Day 0) but were not sustained after 14 days. The lower stream of glycolytic intermediates accumulated significantly throughout the storage period (*p*-values <0.001 versus Day 0). These results are consistent with the findings of Burger et al.; they found that F1,6-BP and DHAP accumulated in the first stage of RBC storage with PAGGGM solution, but the elevations in both metabolites were not sustained during storage period [10]. Intermediates of the non-ox PPP, such as ribose 5-phosphate (R5P) and ribulose 5-phosphate (RU5P), increased sharply within the first 2 days of storage along with a decline in the level of GUO, and then fell abruptly. These metabolites showed similar time series to middle-glycolytic intermediates. Intracellular HX, which is an intermediate of purine salvage pathway, increased progressively, while intracellular ADE was consumed throughout the storage period (Figure 2C).

### Alteration of metabolic flux distributions and metabolic pools during cold storage

**Figure 3:**
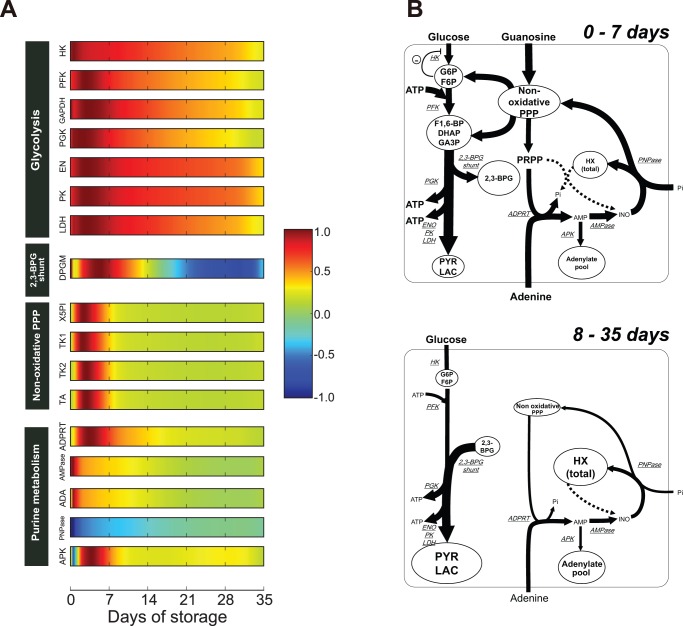
Predicted transition of enzymatic activities and schematic representation of flux distributions in PAGGGM-stored RBCs. *Panel A*: Transition of enzymatic activities. Each enzymatic activity was represented as a normalized value between −1.0 and 1.0 Each value was normalized by an absolute maximum value during 0–35 days of storage simulation. *Panel B*: Schematic representation of metabolic pools and flux distributions in PAGGGM-stored RBCs during 0–7 days of storage (*top panel*) and 8–35 days of storage (*bottom panel*). Total HX represents the sum of intracellular and extracellular HX. Circles and arrows indicate metabolic pools and fluxes, respectively. The width of arrows is proportional to fluxes of metabolic reactions predicted by the model. Abbreviations used in this figure are given in Table 1.

Using the simulation model, we showed the predicted alteration of enzymatic activities of glycolysis, non-ox PPP, and purine salvage pathway (Figure 3A), which facilitated the detection of the imbalance in metabolic pools to be found in PAGGGM-stored RBCs during the storage period. On the basis of the prediction of enzymatic activities and the related metabolite concentrations showed in Figure 3A and Figure 2, we could predict the flux distributions and the flows of the changes in metabolic pools in PAGGGM-stored RBCs (Figure 3B). Although all enzymatic activities seemed to be inactivated by low temperature during cold preservation, there were drastic changes in the patterns of enzymatic activities in our model (Figure 3A). Especially in 0–7 days of storage, both enzymatic activities and intermediates concentrations showed the significant changes compared with the later period. The following sections described a predicted mechanism of the metabolic dynamics in each period.

### Early response in the metabolic dynamics of PAGGGM-stored RBC

Glycolytic enzymes except for hexokinase, non-ox PPP enzymes, adenine phosphoribosyl transferase (ADPRT), and adenylate kinase (APK) were acutely activated during the first 7 days. In particular, non-ox PPP enzymes were significantly activated (∼7.0 times higher than the initial condition) in the earliest stage. These activations seemed to be the result of a GUO-induced increase in non-ox PPP intermediates (Figure 2C). This increase in non-ox PPP through the conversion from GUO was reflected in the increase in the upper glycolytic pool (G6P and F6P) and 5-phosphoribosyl-1-phosphate (PRPP), which are catalyzed by transketolase and PRPP synthetase, respectively. Furthermore, the increases in upper glycolysis and PRPP directly activated PFK and ADPRT, respectively. These results imply that the GUO supplement increased non-ox PPP intermediates and that this effect could be divided into 2 different fluxes, namely, boosting glycolysis and purine salvage pathway in the first 7 days of storage.

In glycolysis, PFK activation is known to increase the pool of middle glycolytic intermediates, which results in maintaining 2,3-BPG [9], [10]. Figure 2 shows that LAC was constantly increased throughout the storage period, although upper glycolysis was activated in first days of storage. This implies that the increased flux of upper and middle glycolysis flows into 2,3-BPG pool, but not into the latter half of glycolysis. On the other hand, both glucose uptake and LAC production rates in the first week were twice as large as those during the rest of period (Figure S5), indicating that the ratio of glucose uptake to LAC production was not changed in all over the storage period. The increase in the upper glycolytic pool repressed hexokinase activity through the enzyme's strong product inhibition effect, which is one of the major ATP-consuming processes in RBCs. Under these circumstances, the increase in the upper glycolytic pool serves as an efficient ATP production system with effective consumption of ATP at glycolysis.

In the purine salvage pathway, the activation of ADPRT through the increase in PRPP promoted an efficient uptake of extracellular ADE. The large amount of ADE resulted in the activation of APK, which catalyzes the synthesis of ADP from AMP, as well as the regeneration of AMP and ATP from ADP. Intracellular ADE constantly decreased during storage from the initial impact of exposure to the ADE-added solution (Figure 2), while the total amount of HX (the sum of intracellular and extracellular HX in the model) increased (Figure 3B). Although the addition of ADE as a source of ATP has been known to prevent the loss of ATP during blood storage [18], ADE might be involved in the accumulation of the end products of the purine salvage pathway, such as HX. In fact, purine nucleoside phosphorylase (PNPase), which catalyzes HX and R1P into inosine, has been constantly reversed to produce HX during storage. These predictions and measured HX accumulations indicated that a part of taken up ADE was supplied to the adenylate pool via AMP, whereas most ADE was eventually converted into HX, which was one of the most accumulated substrates in PAGGGM-stored RBCs.

In summary, flux from GUO through glycolysis contributes to 2,3-BPG accumulation and efficient ATP production in the early storage, also, GUO flux through purine salvage pathway accelerates ADE uptake and subsequently production of ATP and HX.

### Long-term metabolic response in PAGGGM-stored RBC

In spite of the upper glycolytic activation in the beginning of storage, 2,3-BPG declined in the RBCs after 14 days. The decrease in 2,3-BPG was caused by the reversed flux of the 2,3-BPG shunt after 14 days of storage (Figure 3A). Owing to the co-operative inhibition effects of 2,3-BPG synthase activity by protons, the reversed flux was speculated to be due to the decline in pH [19]. This reversed flux of the 2,3-BPG shunt is crucial in maintaining the activities of the latter part of glycolysis and the production of ATP in the latter half of the storage period (Figure 3B, *bottom panel*). In fact, downstream enzymes of 2,3-BPG, such as enolase, pyruvate kinase, and lactate dehydrogenase, kept higher activities, while the enzyme activities of other pathways were significantly repressed after 14 days of storage (Figure 3A, Figure S5). These activations in the latter part of glycolysis are essential for maintaining high ATP levels during the entire storage period, because re-phosphorylation of ADP to produce ATP mainly occurs via this pathway.

### Simulation analysis to identify the role of ADE and GUO in RBC preservation

Our results indicated that the flux flow from GUO causes a global change in RBC metabolism in the initial period of storage, which induces not only the accumulation of 2,3-BPG but also the maintenance of the ATP level in entire storage period. Moreover, ADE contributes to enlarge the adenylate pool size as well as leads to the accumulation of metabolic by-products like HX, which accumulate both intracellular and extracellular space.

**Figure 4:**
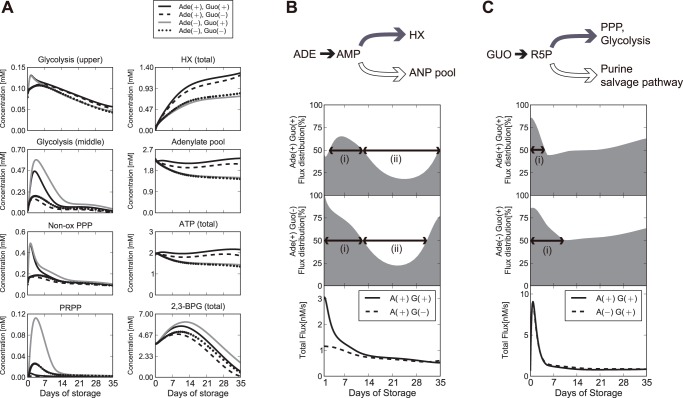
Prediction of adenine- and guanosine-dependent metabolic alterations during cold storage. *Panel A*: Time-related changes of metabolic intermediates with or without adenine (ADE) and guanine (GUO). Simulation with both ADE and GUO (*solid black*, PAGGGM solution), without GUO (*broken black*), without ADE (*solid gray*), and without neither ADE nor GUO (*dotted black*), respectively. Upper glycolysis represents the total concentrations of G6P and F6P; middle glycolysis represents the total of F1,6-BP, DHAP, and GA3P; and non-oxidative pentose phosphate pathway (non-ox PPP) represents the total of R5P, RU5P, X5P, and R1P. The adenylate pool represents the sum concentration of AMP, ADP, and ATP. Total HX represents the sum of intracellular and extracellular HX. *Panel B*: Time-related change of ADE flux distribution through AMP to HX or to the adenylate pool. The ratio of ADE flux distribution into HX (*gray filled curve)* is calculated as *J_HX_*/(*J_HX_* + *J_ANPpool_*) ×100, where *J_HX_* is the sum of enzymatic activities of purine salvage pathway, including AMP deaminase, AMPase, and adenosine kinase, and *J_ANPpool_* is the activity of APK. *1^st^* and *2^nd^ panels* show the ratio of ADE flux distribution into HX in the ADE(+)GUO(+) model and ADE(+)GUO(−) model, respectively. *3^rd^ panel* shows the time-related changes of total ADE flux (*J_HX_* + *J_ANPpool_*) in the ADE(+)GUO(+) (*solid black*) and ADE(+)GUO(−) *(broken black*) model. *Panel C*: Time-related change of GUO flux distribution through R5P to glycolysis or purine salvage pathway. The ratio of GUO flux distribution into glycolysis (*gray filled curves*) is calculated as *J_glycolysis_*/(*J_glycolysis_* + *J_purine_*) ×100, where *J_glycolysis_* is the sum of enzymatic activities of R5PI and TK1 and *J_purine_* is the activity of PRPPsyn. *1^st^* and *2^nd^ panels* show the ratio of GUO flux distribution into glycolysis in the ADE(+)GUO(+) model and ADE(−)GUO(+) model, respectively. *3^rd^ panel* shows the total GUO flux (*J_glycolysis_* + *J_purine_*) in the ADE(+)GUO(+) (*solid black*) and ADE(−)GUO(+) (*broken black*) models.

Next, we performed simulation analysis to clarify the effect of ADE and GUO on the global metabolic change in PAGGGM-stored RBCs. Figure 4A shows the simulation results of the model with and without ADE and/or GUO. The level of the adenylate pool and total amount of ATP and HX were higher in ADE(+) conditions (*black solid line* and *black broken line*) than ADE(−) conditions (Figure 4A). The largest pool sizes of these metabolites were shown in the ADE(+)GUO(+) model. At 35 days of storage, total concentrations of HX and ATP in the ADE(+)GUO(+) model were predicted to be 1.5 times and 1.6 times as much as those of the ADE(−)GUO(−) model, respectively. Whereas, intracellular HX was not significantly accumulated in ADE(+) rather than ADE(−) model owing to the acceleration of HX export by extracelluler ADE (Figure S6). GUO can also contribute to the increase in ATP level, HX level, and the adenylate pool, but these boosting effects were observed only in the ADE(+) condition. These results showed that ADE is the most influential factor to determine the yields of the adenylate pool, ATP, and HX over the storage period. HX is known to react with xanthine oxidase to generate reactive oxygen species [20], and there is ample evidence that the reaction of xanthine oxidase in plasma induces sickle cell anemia [21]. Thus, a large amount of HX in the storage blood pack may therefore be harmful due to an increase in plasma reactive oxygen species after transfusion.

To investigate the fate of ADE within RBCs during long-term storage, the ratio of flux distribution at an AMP branch point was calculated (Figure 4B). Because ADE is metabolized via this branch in RBC metabolism, one fate is into HX (*J_HX_*), which is calculated as the sum of enzymatic activities of purine salvage pathway, including AMP deaminase, AMPase, and adenosine kinase, and another fate is into the adenylate pool (*J_ANPpool_*), which can be mimicked by APK activity. The ratio of flux through HX (%) is calculated as *J_HX_*/(*J_HX_* + *J_ANPpool_*) ×100. This result indicated that more than half of the flux from ADE was not fixed into the adenylate pool but directly passed through purine salvage pathway to supply HX from 2 to 12 days of storage (Figure 4B *1^st^ panel*, (i)). In this period, the total flux from ADE through AMP was quite large (Figure 4B *bottom panel*) in the ADE(+)GUO(+) condition and resulted in the acceleration of HX accumulation during the initial period of storage. After 12 days of storage, flux flow from ADE into the adenylate pool was higher than that into HX (Figure 4B *1^st^ panel*, (ii)). These results imply that the incorporation of ADE into the adenylate pool persisted throughout the storage period. These findings are consistent with the earlier observation of the continued gradual disappearance of ADE-^14^C and the sustained rise in the adenine nucleotide with specific radioactivity in acid citrate dextrose (ACD)-blood during storage [22].

The initial large peak of non-ox PPP appeared only in the GUO(+) condition (*black solid lin*e and *gray solid line*) (Figure 4A). This acute increase in non-ox PPP implies that GUO is taken as a ribose source in non-ox PPP in RBCs. As mentioned above, the flux of the ribose source derived from GUO is partitioned in 2 directions, towards glycolysis and towards purine salvage pathway. The flux through glycolysis increases middle glycolytic intermediates and then results in 2,3-BPG production. In fact, in the models with GUO, glycolytic intermediates and 2,3-BPG accumulate more than in GUO(−) models (Figure 4A). From these results, as well as the previous results, it can be concluded that GUO can boost the 2,3-BPG production via the accumulation of upper glycolytic intermediates, which can trigger PFK activation. The larger NADH/NAD ratio and unchanged LAC production rate in GUO(+) model also suggest that upper glycolytic flux was increased but retained in 2,3-BPG pool in the presence of GUO (Figure S6). Moreover, interestingly, these accumulations are enhanced in the ADE(−) condition (Figure 4A). PRPP, which is located at the gate between purine salvage pathway and non-ox PPP, significantly accumulates under the ADE(−)GUO(+) condition, whereas purine metabolic intermediates such as HX and the adenylate pool do not increase under ADE(−)GUO(+) condition. Taken together with the fact that PRPP reacts with ADE catalyzed by ADPRT, these results suggest that the GUO flux fate is largely controlled by ADE.

To confirm the effect of ADE addition on GUO fate, we also calculated the ratio of flux distribution of GUO at the purine salvage pathway branch point (Figure 4C) with or without ADE. The ratio of GUO flux towards glycolysis (%) at the branch point is calculated as *J_gly_*/(*J_gly_* + *J_purine_*) ×100, where *J_gly_* is the flux into glycolysis, which is the sum of enzymatic activities of transketolase and R5P isomerase, and *J_purine_* represents the flux into purine salvage pathway, which is considered an activity of PRPP synthetase. Regardless of the presence or absence of ADE, flux distribution of GUO into glycolysis was more than 80% at the beginning of the storage period, after which the ratio gradually decreased. This unbalanced flux pattern lasted for only 4 days in the ADE(+) model (Figure 4C *1^st^ panel* (i)), whereas it continued for as long as 11 days in the ADE(−) model (Figure 4C *2^nd^ panel* (i)). These results reveal that ADE is capable of shifting the GUO flux into a purine salvage pathway other than glycolysis. The significant accumulation of inosine monophosphate in ADE(−) also supports that the fate of GUO is strongly dependent on the presence of ADE (Figure S6). Besides, the ADE-induced GUO flux shift to purine salvage pathway results in reducing ribose phosphate in non-ox PPP and an accompanied reduction in 2,3-BPG in PAGGGM-stored RBCs. It is known that the addition of ADE promotes 2,3-BPG depletion [18]. The ADE-induced 2,3-BPG reduction is reported to be caused by favoring 1,3-BPG metabolism via phosphoglycerate kinase (PGK) due to the higher ADP level [22]. The present study also showed that PGK was activated in the ADE(+) model rather than in the ADE(−) model (data not shown). In addition, our simulation result showed that ADE-induced reduction of ribose phosphate also hastens 2,3-BPG depletion, but the addition of GUO can compensate for this depletion.

Our predicted results suggested the coordinating effects of ADE and GUO on RBC metabolism. Both GUO and ADE are necessary for the maintenance and improvement of ATP and 2,3-BPG levels in PAGGGM-stored RBCs. In addition, our results provided unique insights into the intricate regulatory network of energy metabolism by ADE and GUO during cold storage of RBCs. ADE acts as a source of ATP, but it reduces 2,3-BPG. GUO is used as a source of ribose phosphate, and it induces PFK activation, which can result in compensating for the depletion of 2,3-BPG by ADE. The fate of GUO between the non-ox PPP and purine salvage pathway is largely controlled by ADE. These findings illustrated that the metabolic trade-offs between ATP and 2,3-BPG, which are commonly observed in conventional blood storage methods, can be offset by ADE and GUO in PAGGGM-stored RBCs.

**Figure 5:**
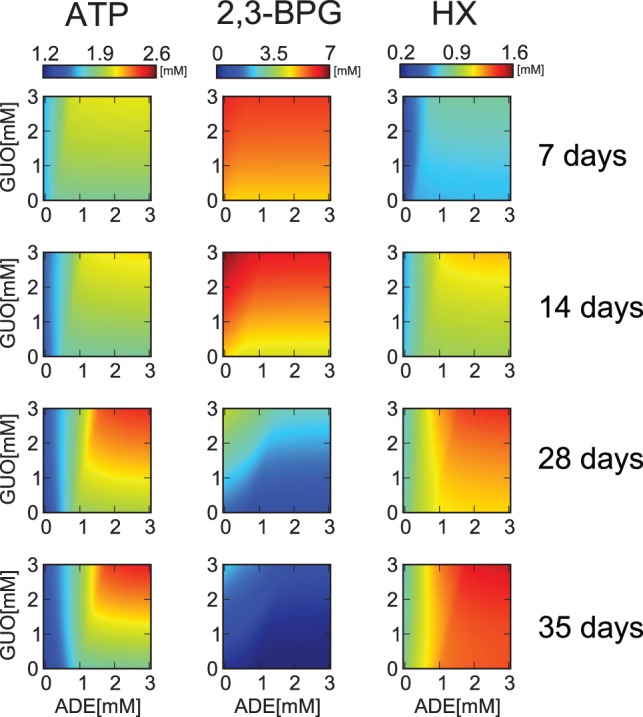
Prediction of intracellular metabolite levels depending on the combination of initial adenine and guanine concentrations. Predicted concentrations of ATP (*left column*), 2,3-BPG (*center column*), and total HX (*right column*) at 7, 14, 28, and 35 days after storage in various combinations of ADE and GUO concentrations. In each panel, the x and y axes represent initial concentration of ADE and GUO in the model, respectively. The initial setting of ADE and GUO varied between 0 to 3 mM.

Simulation approaches can unravel mechanisms of such a complicated system, networks, and regulations, including the coordinated effects of metabolites. Precise and large-scale kinetic modeling is also useful as a tool for predicting the ideal combination of solution additives for longer blood storage or giving insights on the appropriate use of the different storage methods, depending on the patients' condition. To determine the optimal amount of ADE and GUO as storage-solution additives, ATP, 2,3-BPG, and HX levels at various combinations of ADE and GUO concentrations after 7, 14, 28, and 35 days of storage were predicted (Figure 5). Higher ADE and GUO concentrations maintained higher ATP, unless ADE was below 1 mM, in which case ATP remarkably decreased with storage time, regardless of GUO concentration. As expected from the flux distribution analysis of GUO, the 2D plots also showed the effect of the high concentration of GUO in 2,3-BPG maintenance and the inhibitory effect of ADE on it. HX level was predicted to be mainly determined by ADE and less affected by GUO, especially before 2 weeks of storage.

It is important to understand metabolic dynamics of stored RBCs comprehensively to estimate the benefits and side effects of solution additives used for blood preservation. In recent years, proteomics and metabolomics approaches have been applied to investigate storage lesions in RBCs [23], [24], [25], [26]. However, it is difficult to interpret large sets of “omics” data for the extraction of essential information about biological systems. *In silico* simulation can help to handle a massive amount of omics data for the extraction of novel information on metabolic dynamics in RBC storage.

### Limitation of the PAGGGM-stored RBC model

Some key biochemical traits have yet to be integrated in the PAGGGM-stored RBC model, such as autonomic regulation of intracellular pH including acidotic shift caused by LAC accumulation, cell volume regulation and osmoregulation due to a change in ion balance. These are tightly connected to the physiological aspects of RBCs which are not considered, for example, loss of membrane plasticity, and the associated alterations in cell shape and rheology [4], which are also assessed in relation with metabolic injury like the depletion of ATP and anti-oxidative metabolites.

Several studies proposed physiological models of human RBCs on various aspects; pH changes in erythrocyte with respect to ion and osmotic balance [27], membrane plasticity and shape transformation by ATP-induced cytoskeletal disorders [28], erythrocyte rheology and its mechanics [29], and blood viscosity in relation to shear stress and hematocrit [30]. In spite of the difficulties in the integration of different types of simulation models across multiple layers of time- and spatial- scale, the recent advances in cellular simulators have made it feasible to model various processes at a whole-cell level [31], [32]. Integrating these physiological models with the precise metabolic pathway model such as that used in this study enables to predict more realistic cell properties and will accelerate experimental discovery towards the novel blood storing methodologies.

## Conclusions

In this study, we constructed a large-scale metabolic model of RBCs preserved in a recently developed blood storage solution named PAGGGM, which can maintain both ATP and 2,3-BPG during 5 weeks. The model was validated by comparison with time-series metabolomics data from CE-TOFMS experiments during long-term cold storage of RBCs. From the simulation results of metabolites and enzymatic activities, we showed that metabolic dynamics of PAGGGM-stored RBCs were drastically changed during storage period by the supplementation levels of ADE and GUO. The model demonstrated that the coordinated action of these additives was required for dynamically maintaining levels of both ATP and 2,3-BPG under cold storage conditions. GUO is mainly used as a source of ribose phosphate in non-ox PPP and can boost the production of both ATP and 2,3-BPG depending on PFK activation. ADE can enlarge the adenylate pool, which results in keeping ATP levels high but directly causes HX accumulation. ADE also has an important role in the determination of GUO fate under PAGGGM-RBC preservation conditions.

To our knowledge, this is the first *in silico* study to provide a detailed explanation of the underlying mechanism of metabolic lesions and their compensation by solution additives in RBCs under long-term cold storage, as well as of the roles and fates of additives in the context of metabolic benefits and possible side effects.

## Supporting Information

Figure S1 **Measured and simulated time-series of guanosine consumptions.** Guanosine consumptions in PAGGGM-stored RBC measured by CE-TOFMS (i) and described by the simulation model (ii) were displayed. In each panel, time-series of guanosine (ratios) were calculated as a normalized to Day 0 concentration, respectively. CE-TOFMS data were expressed as means ± SD of 6 separate experiments.
(EPS)

Figure S2 **Time-series of glycolytic intermediates predicted by cold-stored RBC models with original and re-tuned parameters.** Measured and predicted time courses of intermediates in glycolysis in MAP-stored RBC (A) and PAGGGM-stored RBC (B) were illustrated. In simulation results of each panel, the time-series were predicted by original parameters in ref. [9] (*solid black line*), where three groups (Na^+^/K^+^ pump, purine salvage and other reactions) of reaction activities were set as 0.6%, 24%, 3.4% of the base model (37°C), and predicted by re-tuned parameters (*broken black line*), where those were set as 0.1%, 25%, 3.0%, respectively. The parameter estimation was performed by real number genetic algorithm to fit with 8 points time-series of ATP and 2,3-BPG in cold-stored RBCs preserved in Mannitol-Adenine-Phosphate (MAP) solution. The parameters were searched within their feasible ranges which we provided in previous study in ref. [9]. The PAGGGM-stored model with the re-tuned parameters for reaction activities (0.1%, 25.0%, and 3.0%) showed better agreement with CE-TOFMS measurements than the model with the original parameters included in MAP model.
(EPS)

Figure S3 **Robustness of the PAGGGM-stored RBC model against kinetic parameter changes.** The sensitivity analysis of all kinetic parameters against the dynamics of ATP and 2,3-BPG was carried out. Time courses of ATP and 2,3-BPG were plotted with 10% decrease (A and B) and with 10% increase in each parameter (C and D). In the plots, black solid lines show the original behaviors with no change in parameters. The sensitivities of ATP- and 2,3-BPG- time courses to the change of each kinetic parameter were not quite large (data not shown). This figure showed the changes of dynamics of ATP and 2,3-BPG of largest five parameters.
(EPS)

Figure S4 **Sensitivity of metabolic intermediates dynamics to the rate constants of guanosine phosphorylation.** The rate constant (*k*) of guanosine phosphorylation process was varied from 1e+6 to 1e+9 in the model. *k* was changed to 1e+6 (*broken black*), 1e+7 (*solid gray*), 1e+8 (*solid black*; default value of the model), and 1e+9 (*dotted black* ).
(EPS)

Figure S5 **The time-dependent changes in substrates uptake and production in PAGGGM-stored RBC.** Glucose (GLC), adenine (ADE) and guanosine (GUO) uptake rates and lactate (LAC), pyruvate (PYR) and hypoxanthine (HX) production rates are shown. In each panel, the uptake/production rates during 0–7 days and 8–35 days of storage are shown, respectively. Both glucose uptake and LAC production rates in the first week were twice as large as those during the rest of period, supporting that the ratio of glucose uptake to LAC production was not changed in all over the storage period. Besides, the large increase in PYR production rate was observed during 8–35 days of storage, indicating that ATP was continuously produced in the latter half period. As a result, ATP was maintained at a suitable level throughout the storage period.
(EPS)

Figure S6 **Predicted adenine- and guanosine-dependent metabolic alterations during cold storage.** Time-related changes of metabolic intermediates with or without adenine (ADE) and guanosine (GUO). Abbreviations are given in Table 1. NADH/NAD and NADPH/NADP showed redox ratio of each co-enzyme, respectively.
(EPS)

Model S1 **PAGGGM-stored RBC model written in SBML format.** This SBML model can be imported to and run with COPASI 4.8 (Build 35). The calculation accuracy of the SBML model was confirmed with the E-Cell model.
(XML)

Table S1 **Comparison of based and cold-stored RBC metabolic models.**
(PDF)

Text S1 **Detailed description of PAGGGM-stored RBC model and parameter settings.**
(PDF)
