# Supplementary material for: Dynamic Simulation and Metabolome Analysis of Long-Term Erythrocyte Storage in Adenine–Guanosine Solution
Source: PLoS One. 2013 Aug 16;8(8):e71060. doi: 10.1371/journal.pone.0071060 (PMC3796775; doi:10.1371/journal.pone.0071060)
Supplement: Table S1 — Comparison of based and cold-stored RBC metabolic models. (PDF) [file pone.0071060.s008.pdf]

| | Base model (37℃) | MAP model (4℃) | PAGGGM model (4℃) |
|---|---|---|---|
| Enzymatic activities | 100% | 24% | 25% |
| Purine metabolism activities | 100% | 34% | 30% |
| Sodium/pottasium pump activity | 100% | 6.0% | 1.0% |
| Extracellular glucose | 5 mM (fixed) | 40 mM | 47.5 mM |
| Extracellular adenine | 0.015 mM (fixed) | 1.04 mM | 1.44 mM |
| Extracellular inorganic phosphate | 1.1 mM (fixed) | 6 mM | 16 mM |
| Extracellular sodium ion | 135 mM (fixed) | 106 mM | 64 mM |
| Extracellular guanosine | - | - | 1.44 mM |
| Guanosine phosphorylation | - | - | First-order kinetics |
| pH setting | pH = 7.2 (fixed) | Initial pH = 6.95, $\mathrm{pH(t)} = at + 6.95$ $a = -8.66 \times 10^{-8}$ | Initial pH = 7.62, $\mathrm{pH(t)} = at^2 + bt + 7.62$ $a = 8.79 \times 10^{-14}$ $b = -6.01 \times 10^{-7}$ |
| State of hemoglobin | Transition between T- and R-state in response to various parameters; $pO_2$, $pCO_2$, pH, temperature and 2,3-BPG | R-state | R-state |
