# Supplementary material for: Dynamic Simulation and Metabolome Analysis of Long-Term Erythrocyte Storage in Adenine–Guanosine Solution
Source: PLoS One. 2013 Aug 16;8(8):e71060. doi: 10.1371/journal.pone.0071060 (PMC3796775; doi:10.1371/journal.pone.0071060)
Supplement: Text S1 — Detailed description of PAGGGM-stored RBC model and parameter settings. (PDF) [file pone.0071060.s009.pdf]

Supporting Information (Nishino *et al.*)
Text S1.

1. Detailed description of PAGGGM-stored RBC model.

The main body of the model is based on the published human erythrocyte metabolic model [1]. All reactions expressed in the model are shown below:

1-1. Kinetic equation and parameters used in the model

Abbreviation of all reactions and reactants are corresponding to those shown in Table 1 in the main text. e_t denotes enzyme concentration.

HK

$$v = \frac{e_t\left(\frac{theK_{catf}\mathrm{AB}}{K_{i,\mathrm{B}}K_{m,\mathrm{A}}} - \frac{theK_{catr}\mathrm{PQ}}{K_{i,\mathrm{Q}}K_{m,\mathrm{P}}}\right)}{1+\frac{\mathrm{A}}{K_{i,\mathrm{A}}}+\frac{\mathrm{B}}{K_{i,\mathrm{B}}}+\frac{\mathrm{AB}}{K_{i,\mathrm{B}}K_{m,\mathrm{A}}}+\frac{\mathrm{P}}{K_{i,\mathrm{P}}}+\frac{\mathrm{Q}}{K_{i,\mathrm{Q}}}+\frac{\mathrm{PQ}}{K_{i,\mathrm{Q}}K_{m,\mathrm{Q}}}+\sum_{j=1}^{4}\frac{\mathrm{I}_j\mathrm{B}}{K'_{i,\mathrm{I}j}K_{i,\mathrm{B}}}} \quad \text{(S1)}$$

$$theK_{catf} = \frac{1.662k_{catf}}{\left(1+\frac{10^{-\mathrm{pH}}}{10^{-7.02}}+\frac{10^{-9.55}}{10^{-\mathrm{pH}}}\right)} \quad \text{(S2)}$$

$$theK_{catr} = \frac{1.662k_{catr}}{\left(1+\frac{10^{-\mathrm{pH}}}{10^{-7.02}}+\frac{10^{-9.55}}{10^{-\mathrm{pH}}}\right)} \quad \text{(S3)}$$

Symbols: A, MgATP; B, GLC; P, G6P; Q, MgADP; I, Pi, 2,3-BPG and GDP

Parameter	Value
e_t (M)	2.50E–08
$K_{m.\mathrm{MgADP}}$, $K_{i.\mathrm{MgADP}}$ (M)	1.00E–03
$K_{m,\mathrm{MgATP}}$, $K_{i,\mathrm{MgATP}}$ (M)	1.00E–03
$K'_{i.2.3\mathrm{BPG}}$ (M)	2.70E–03
$K'_{i.\mathrm{GSH}}$ (M)	3.00E–03
$K'_{i.\mathrm{GDP}}$ (M)	1.00E–05
$K'_{i.\mathrm{G6P}}$ (M)	1.00E–05
$K_{i.\mathrm{GLC}}$ (M)	4.70E–05
$K_{m.\mathrm{G6P}}$, $K_{i.\mathrm{G6P}}$ (M)	4.70E–05
k_{catf} (s^{-1})	180
k_{catr} (s^{-1})	1.16

Parameter values were taken from [2] or adjusted by reference to observed data corrected by the literature.

Text S1.

PGI, TPI, PGM

$$v = \frac{e_t\left(\frac{k_{catf}\mathrm{A}}{K_{m,\mathrm{A}}} - \frac{k_{catr}\mathrm{P}}{K_{m,\mathrm{P}}}\right)}{1 + \frac{\mathrm{A}}{K_{m,\mathrm{A}}} + \frac{\mathrm{P}}{K_{m,\mathrm{P}}}} \quad \text{(S4)}$$

Symbols: (PGI) A,G6P; P, F6P (TPI) A, DHAP; P, GA3P (PGM) A, 3PG; P, 2PG

Parameter	Value
PGI	
e_t (M)	2.18E−07
$K_{m\text{.G6P}}$ (M)	1.81E−04
$K_{m\text{.F6P}}$ (M)	7.10E−05
k_{catf} (s^{-1})	1470
k_{catr} (s^{-1})	1760
PGM	
e_t (M)	4.10E−07
$K_{m\text{.2PG}}$ (M)	4.60E−05
$K_{m\text{.3PG}}$ (M)	1.68E−04
k_{catf} (s^{-1})	795
k_{catr} (s^{-1})	714
TPI	
e_t (M)	1.14E−06
$K_{m\text{.DHAP}}$ (M)	1.62E−04
$K_{m\text{.GA3P}}$ (M)	4.46E−04
k_{catf} (s^{-1})	1280
k_{catr} (s^{-1})	14560

Parameter values were taken from [2] or adjusted by reference to observed data corrected by the literature.

Supporting Information (Nishino *et al.*)
Text S1.

PFK

$$v = \frac{e_t\left(\frac{k_{catf}\mathrm{AB}}{K_{mR,\mathrm{A}}K_{mR,\mathrm{B}}} - \frac{k_{catr}\mathrm{PQ}}{K_{mR,\mathrm{P}}K_{mR,\mathrm{Q}}}\right)}{1+\frac{\mathrm{A}}{K_{mR,\mathrm{A}}}+\frac{\mathrm{B}}{K_{mR,\mathrm{B}}}+\frac{\mathrm{AB}}{K_{mR,\mathrm{A}}K_{mR,\mathrm{B}}}+\frac{\mathrm{P}}{K_{mR,\mathrm{P}}}+\frac{\mathrm{Q}}{K_{mR,\mathrm{Q}}}+\frac{\mathrm{PQ}}{K_{mR,\mathrm{P}}K_{mR,\mathrm{Q}}}} \times \rho \qquad \text{(S5)}$$

$$\rho = \frac{1}{1+L_{PFK}} \qquad \text{(S6)}$$

$$L_{PFK} = \frac{\left(\frac{10^{-\mathrm{pH}}}{K_a}\right)^n \left(1+\frac{\mathrm{ATP}}{K_{T,\mathrm{ATP}}}\right)^4 \left(1+\frac{\mathrm{Mg}^{2+}}{K_{T,\mathrm{Mg}^{2+}}}\right)^4 \left(1+\frac{\mathrm{2,3BPG}}{K_{T,\mathrm{2,3BPG}}}\right)^4}{\left(1+\frac{\mathrm{F6P}}{K_{mR,\mathrm{F6P}}}+\frac{\mathrm{F16BP}}{K_{mR,\mathrm{F16BP}}}\right)^4 \left(1+\frac{\mathrm{AMP}}{K_{R,\mathrm{AMP}}}\right)^4 \left(1+\frac{\mathrm{Pi}}{K_{R,\mathrm{Pi}}}\right)^4 \left(1+\frac{\mathrm{GDP}}{K_{R,\mathrm{GDP}}}\right)^4} \qquad \text{(S7)}$$

Symbols: A, MgATP; B, F6P; P, F1,6BP; Q, MgADP

Parameter	Value
e_t (M)	1.10E−07
$K_{R,\mathrm{AMP}}$ (M)	3.50E−05
$K_{R,\mathrm{GDP}}$ (M)	1.51E−05
$K_{R,\mathrm{Pi}}$ (M)	4.31E−04
$K_{T,\mathrm{ATP}}$ (M)	9.80E−06
$K_{T,\mathrm{2,3BPG}}$ (M)	1.44E−03
$K_{T,\mathrm{Mg}^{2+}}$ (M)	4.40E−04
$K_{mR,\mathrm{F1,6BP}}$ (M)	4.20E−04
$K_{mR,\mathrm{F6P}}$ (M)	2.70E−04
$K_{mR,\mathrm{MgADP}}$ (M)	5.40E−04
$K_{mR,\mathrm{MgATP}}$ (M)	6.80E−05
n	2
K_a	8.91E−08
k_{catf} (s^{-1})	822
k_{catr} (s^{-1})	36

Parameter values were taken from [2] or adjusted by reference to observed data corrected by the literature.

Supporting Information (Nishino *et al.*)
Text S1.

ALD

$$v=\frac{e_t\left(\frac{k_{catf}\mathrm{A}}{K_{m,\mathrm{A}}}-\frac{k_{catr}\mathrm{PQ}}{K_{i,\mathrm{Q}}K_{m,\mathrm{P}}}\right)}{1+\frac{\mathrm{I}}{K_{i,\mathrm{I}}}+\frac{\mathrm{A}}{K_{m,\mathrm{A}}}+\frac{K_{m,\mathrm{A}}\mathrm{P}}{K_{m,\mathrm{P}}K_{i,\mathrm{Q}}}\left(1+\frac{\mathrm{I}}{K_{i,\mathrm{I}}}\right)+\frac{\mathrm{Q}}{K_{i,\mathrm{Q}}}+\frac{K_{m,\mathrm{Q}}\mathrm{AP}}{K_{i,\mathrm{A}}K_{m,\mathrm{P}}K_{i,\mathrm{Q}}}+\frac{\mathrm{PQ}}{K_{i,\mathrm{Q}}K_{m,\mathrm{P}}}} \quad \text{(S8)}$$

Symbols: A, F1,6BP; P, GA3P; Q, DHAP;I, 2,3BPG

Parameter	Value
e_t (M)	3.70E−07
$K_{m,\mathrm{F1,6BP}}$ (M)	1.65E−05
$K_{i,\mathrm{F1,6BP}}$ (M)	1.98E−05
$K_{m,\mathrm{DHAP}}$ (M)	3.50E−05
$K_{i,\mathrm{DHAP}}$ (M)	1.10E−05
$K_{m,\mathrm{GA3P}}$ (M)	1.90E−04
$K_{i,\mathrm{2,3BPG}}$ (M)	1.50E−03
k_{catf} (s^{-1})	68
k_{catr} (s^{-1})	234

Parameter values were taken from [2] or adjusted by reference to observed data corrected by the literature.

GAPDH

$$v=\frac{e_t\left(\frac{k_{catf}\mathrm{ABC}}{K_{m,\mathrm{A}}K_{i,\mathrm{B}}K_{i,\mathrm{C}}}-\frac{10^{-\mathrm{pH}}k_{catr}\mathrm{PQH}}{10^{-7.2}K_{i,\mathrm{P}}K_{m,\mathrm{Q}}}\right)}{GAPDH_{rd}} \quad \text{(S9)}$$

$$GAPDH_{rd}=\frac{\mathrm{C}}{K_{i,\mathrm{C}}}\left(1+\frac{\mathrm{C}}{K'_{i,\mathrm{C}}}\right)+\frac{\mathrm{P}}{K_{i,\mathrm{P}}}\left(1+\frac{\mathrm{C}}{K'_{i,\mathrm{C}}}\right)+\frac{10^{-\mathrm{pH}}K_{m,\mathrm{P}}\mathrm{Q}}{10^{-7.2}K_{i,\mathrm{P}}K_{m,\mathrm{Q}}}+$$
$$\frac{K_{m,\mathrm{C}}\mathrm{AB}}{K_{m,\mathrm{A}}K_{i,\mathrm{B}}K_{i,\mathrm{C}}}+\frac{\mathrm{AC}}{K_{i,\mathrm{A}}K_{i,\mathrm{C}}}+\frac{\mathrm{BC}}{K_{i,\mathrm{B}}K_{i,\mathrm{C}}}\left(1+\frac{\mathrm{C}}{K'_{i,\mathrm{C}}}\right)+\frac{\mathrm{AP}}{K_{i,\mathrm{A}}K_{i,\mathrm{P}}}+\frac{10^{-\mathrm{pH}}K_{m,\mathrm{P}}\mathrm{BQ}}{10^{-7.2}K_{i,\mathrm{B}}K_{i,\mathrm{P}}K_{m,\mathrm{Q}}}+$$
$$\frac{10^{-\mathrm{pH}}\mathrm{CQ}}{10^{-7.2}K_{i,\mathrm{C}}K_{i,\mathrm{Q}}}+\frac{10^{-\mathrm{pH}}\mathrm{PQ}}{10^{-7.2}K_{i,\mathrm{P}}K_{m,\mathrm{Q}}}+\frac{\mathrm{ABC}}{K_{m,\mathrm{A}}K_{i,\mathrm{B}}K_{i,\mathrm{C}}}+\frac{K_{m,\mathrm{C}}\mathrm{ABP}}{K_{i,\mathrm{C}}K_{m,\mathrm{A}}K_{i,\mathrm{B}}K'_{i,\mathrm{P}}}+$$
$$\frac{10^{-\mathrm{pH}}\mathrm{BCQ}}{10^{-7.2}K_{i,\mathrm{B}}K_{i,\mathrm{C}}K_{i,\mathrm{Q}}}+\frac{10^{-\mathrm{pH}}K_{m,\mathrm{P}}\mathrm{BPQ}}{10^{-7.2}K_{i,\mathrm{P}}K_{m,\mathrm{Q}}K_{i,\mathrm{B}}K'_{i,\mathrm{P}}}$$

(S10)

Symbols: A, NAD^+; B, Pi; C, GA3P; P, 1,3BPG; Q, NADH

Supporting Information (Nishino *et al.*)
Text S1.

Parameter	Value
e_t (M)	7.66E–06
$K_{m.NAD^+}$ (M)	4.50E–05
$K_{i.NAD^+}$ (M)	4.50E–05
$K_{m.1.3BPG}$ (M)	3.30E–06
$K_{i.GA3P}$ (M)	6.50E–02
$K_{i.1.3BPG}$ (M)	1.00E–02
$K_{m.Pi}$ (M)	3.16E–03
$K_{i.Pi}$ (M)	3.16E–03
$K_{m.GA3P}$ (M)	9.50E–05
$K'_{i.GA3P}$ (M)	3.10E–05
$K_{i.NADH}$ (M)	1.00E–05
$K'_{i.1.3BPG}$ (M)	1.00E–06
$K_{m.NADH}$ (M)	3.30E–06
k_{catf} (s^{-1})	232
k_{catr} (s^{-1})	2765

Parameter values were taken from [2] or adjusted by reference to observed data corrected by the literature.

PGK

$$v = \frac{e_t\left(\dfrac{k_{catf}\mathrm{AB}}{K_{m,\mathrm{A}}K_{i,\mathrm{B}}} - \dfrac{k_{catr}\mathrm{PQ}}{K_{i,\mathrm{Q}}K_{m,\mathrm{P}}}\right)}{1+\dfrac{\mathrm{A}}{K_{i,\mathrm{A}}}+\dfrac{\mathrm{B}}{K_{i,\mathrm{B}}}+\dfrac{\mathrm{AB}}{K_{i,\mathrm{B}}K_{m,\mathrm{A}}}+\dfrac{\mathrm{P}}{K_{i,\mathrm{P}}}+\dfrac{\mathrm{Q}}{K_{i,\mathrm{Q}}}+\dfrac{\mathrm{PQ}}{K_{i,\mathrm{Q}}K_{m,\mathrm{P}}}} \quad \text{(S11)}$$

Symbols: A, 1,3BPG; B, MgADP; P, 3PG; Q, MgATP

Parameter	Value
e_t (M)	2.74E–06
$K_{i.1.3\text{-}BPG}$ (M)	1.60E–06
$K_{i.MgADP}$ (M)	8.00E–05
$K_{i.MgATP}$ (M)	1.30E–04
$K_{i.3PG}$ (M)	2.05E–04
$K_{m.1.3\text{-}BPG}$ (M)	2.00E–06
$K_{m.3PG}$ (M)	1.1E–03
k_{catf} (s^{-1})	2290
k_{catr} (s^{-1})	917

Parameter values were taken from [2] or adjusted by reference to observed data corrected by the literature.

Supporting Information (Nishino *et al.*)
Text S1.

EN

$$v=\frac{e_t\left(\frac{k_{catf}\mathrm{AB}}{K_{m,\mathrm{A}}K_{i,\mathrm{B}}}-\frac{k_{catr}\mathrm{PQ}}{K_{i,\mathrm{Q}}K_{m,\mathrm{P}}}\right)}{1+\frac{\mathrm{A}}{K_{i,\mathrm{A}}}+\frac{\mathrm{B}}{K_{i,\mathrm{B}}}+\frac{\mathrm{AB}}{K_{i,\mathrm{B}}K_{m,\mathrm{A}}}+\frac{\mathrm{Q}}{K_{i,\mathrm{Q}}}+\frac{\mathrm{PQ}}{K_{i,\mathrm{Q}}K_{m,\mathrm{P}}}} \qquad \text{(S12)}$$

Symbols: A, 2PG; B, Mg^{2+}; P, Mg^{2+}; Q, PEP

Parameter	Value
e_t (M)	2.20E−07
$K_{i,\mathrm{Mg}^{2+}}$ (M)	4.60E−04
$K_{i,\mathrm{PEP}}$ (M)	3.10E−04
$K_{i,\mathrm{2PG}}$ (M)	1.40E−04
$K_{m,\mathrm{Mg}^{2+}}$ (M)	4.60E−05
$K_{m,\mathrm{2PG}}$ (M)	1.40E−04
k_{catf} (s^{-1})	190
k_{catr} (s^{-1})	50

Parameter values were taken from [2] or adjusted by reference to observed data corrected by the literature.

PK

$$v=\frac{e_t\left(\frac{k_{catf}\mathrm{AB}}{K_{mR,\mathrm{A}}K_{mR,\mathrm{B}}}-\frac{k_{catr}\mathrm{PQ}}{K_{mR,\mathrm{P}}K_{mR,\mathrm{Q}}}\right)}{1+\frac{\mathrm{A}}{K_{mR,\mathrm{A}}}+\frac{\mathrm{B}}{K_{mR,\mathrm{B}}}+\frac{\mathrm{AB}}{K_{mR,\mathrm{A}}K_{mR,\mathrm{B}}}+\frac{\mathrm{P}}{K_{mR,\mathrm{P}}}+\frac{\mathrm{Q}}{K_{mR,\mathrm{Q}}}+\frac{\mathrm{PQ}}{K_{mR,\mathrm{P}}K_{mR,\mathrm{Q}}}}\times\rho \qquad \text{(S13)}$$

$$\rho=\frac{1}{1+L_{PK}} \qquad \text{(S14)}$$

$$L_{PK}=\frac{\left(\frac{10^{-6.8}}{10^{-\mathrm{pH}}}\right)\left(1+\frac{\mathrm{ATP}}{K_{T,\mathrm{ATP}}}\right)^4}{\left(1+\frac{\mathrm{PEP}}{K_{mR,\mathrm{PEP}}}+\frac{\mathrm{PYR}}{K_{mR,\mathrm{PYR}}}\right)^4\left(1+\frac{\mathrm{F1,6BP}}{K_{R,\mathrm{F1,6BP}}}+\frac{\mathrm{GDP}}{K_{R,\mathrm{GDP}}}\right)^4} \qquad \text{(S15)}$$

Symbols: A, MgADP; B, PEP; P, PYR; Q, MgATP.

Parameter	Value
e_t (M)	8.70E−08
$K_{R.\mathrm{F1.6BP}}$ (M)	5.00E−06
$K_{R.\mathrm{GDP}}$ (M)	1.00E−04
$K_{R.\mathrm{MgADP}}$ (M)	4.74E−04
$K_{R.\mathrm{MgATP}}$ (M)	3.00E−03
$K_{mR.\mathrm{PEP}}$ (M)	2.25E−04
$K_{mR.\mathrm{PYR}}$ (M)	2.00E−03
$K_{T.\mathrm{ATP}}$ (M)	3.39E−03
k_{catf} (s^{-1})	1386
k_{catr} (s^{-1})	3.26

Parameter values were taken from [2] or adjusted by reference to observed data corrected by the literature.

LDH

$$v = \frac{e_t\left(\dfrac{k_{catf}\mathrm{AB}}{K_{i,\mathrm{A}}K_{m,\mathrm{B}}} - \dfrac{k_{catr}\mathrm{PQ}}{K_{i,\mathrm{Q}}K_{m,\mathrm{P}}}\right)}{LDH_{rd}} \qquad \text{(S16)}$$

$$LDH_{rd} = \left(1+\frac{K_{m,\mathrm{A}}\mathrm{B}}{K_{i,\mathrm{A}}K_{m,\mathrm{B}}}+\frac{K_{mQ}\mathrm{P}}{K_{mP}K_{iQ}}\right)\left(1+\frac{\mathrm{B}}{K'_{i,\mathrm{B}}}\right)+\frac{\mathrm{A}}{K_{i,\mathrm{A}}}+\frac{\mathrm{Q}}{K_{iQ}}+\frac{\mathrm{AB}}{K_{i,\mathrm{A}}K_{m,\mathrm{B}}}+ \frac{K_{m,\mathrm{Q}}\mathrm{AP}}{K_{i,\mathrm{A}}K_{m,\mathrm{P}}K_{i,\mathrm{Q}}}+\frac{K_{m,\mathrm{A}}\mathrm{BQ}}{K_{i,\mathrm{A}}K_{m,\mathrm{B}}K_{i,\mathrm{Q}}}+\frac{\mathrm{PQ}}{K_{i,\mathrm{Q}}K_{m,\mathrm{P}}}+\frac{\mathrm{ABP}}{K_{i,\mathrm{A}}K_{m,\mathrm{B}}K_{i,\mathrm{P}}}+\frac{\mathrm{BPQ}}{K_{i,\mathrm{B}}K_{m,\mathrm{P}}K_{i,\mathrm{Q}}} \qquad \text{(S17)}$$

Symbols: A, NADH; B, PYR; P, NAD^{+}; Q, LAC

Parameter	Value
e_t (M)	3.43E–06
$K_{m.\mathrm{NADH}}$ (M)	8.44E–06
$K_{i.\mathrm{NADH}}$ (M)	2.45E–06
$K_{m.\mathrm{NAD}^+}$ (M)	1.07E–04
$K_{i.\mathrm{NAD}^+}$ (M)	5.03E–04
$K_{m.\mathrm{PYR}}$ (M)	1.37E–04
$K_{i.\mathrm{PYR}}$ (M)	2.28E–04
$K'_{i.\mathrm{PYR}}$ (M)	1.01E–04
$K_{m.\mathrm{LAC}}$ (M)	1.07E–03
$K_{i.\mathrm{LAC}}$ (M)	7.33E–03
k_{catf} (s^{-1})	458
k_{catr} (s^{-1})	115

Parameter values were taken from [2] or adjusted by reference to observed data corrected by the literature.

LDHP (NADPH dependent)

$$v = \frac{e_t\left(\dfrac{k_{catf}\mathrm{AB}}{K_{m,\mathrm{A}}K_{m,\mathrm{B}}} - \dfrac{k_{catr}\mathrm{PQ}}{K_{m,\mathrm{P}}K_{m,\mathrm{Q}}}\right)}{1+\dfrac{\mathrm{B}}{K_{m,\mathrm{B}}}+\dfrac{\mathrm{Q}}{K_{m,\mathrm{Q}}}} \qquad \text{(S18)}$$

Symbols: A, NADPH; B, PYR; P, $NADP^+$; Q, LAC

Parameter	Value
$e_t\,k_{catf}\,/\,K_{m.\mathrm{NADPH}}$ (s^{-1})	3.46E–03
$e_t\,k_{catr}\,/\,K_{m.\mathrm{NADP}}$ (s^{-1})	5.43E–07
$K_{m.\mathrm{PYR}}$ (M)	4.14E–04
$K_{m.\mathrm{LAC}}$ (M)	4.14E–04

Parameter values were taken from [2] or adjusted by reference to observed data corrected by the literature.

Supporting Information (Nishino *et al.*)
Text S1.

DPGM, DPGase (2,3-BPG shunt)

DPGM:

$$v = \frac{e_t(N_1\mathrm{AB}+N_2\mathrm{AC}+N_3\mathrm{AH})}{D_1\mathrm{A}+D_2\mathrm{B}+D_3\mathrm{C}+D_4\mathrm{D}+D_5\mathrm{H}+D_6\mathrm{AB}+D_7\mathrm{AC}+D_8\mathrm{AH}+D_9\mathrm{BD}+D_{10}\mathrm{CD}+D_{11}\mathrm{DH}} \quad \text{(S19)}$$

DPGase:

$$v = \frac{e_t(N_3\mathrm{AH}+N_7\mathrm{DH}+N_{11}\mathrm{DH})}{D_1\mathrm{A}+D_2\mathrm{B}+D_3\mathrm{C}+D_4\mathrm{D}+D_5\mathrm{H}+D_6\mathrm{AB}+D_7\mathrm{AC}+D_8\mathrm{AH}+D_9\mathrm{BD}+D_{10}\mathrm{CD}+D_{11}\mathrm{DH}}$$

(S20)

$N_1 = k_1k_{12}(k_{15}+k_{16})k_3k_4(k_{10}+k_7)k_8$
$N_2 = k_1k_{10}k_{12}(k_{15}+k_{16})k_3k_6(k_5+k_8)$
$N_3 = k_1k_{14}k_{16}k_3(k_{10}(k_{12}(k_5+k_8)+k_5k_9)+k_7(k_{11}(k_5+k_8)+$
$k_{12}(k_5+k_8)+k_5k_9))$
$N_4 = k_{13}k_{14}k_{16}(k_2+k_3)(k_{11}k_7(k_5+k_8)+k_5(k_{10}+k_7)k_9)$
$N_5 = k_1k_{11}(k_{15}+k_{16})k_3k_4k_7k_8$
$N_6 = k_1k_{10}(k_{15}+k_{16})k_3k_6(k_{12}(k_5+k_8)+k_5k_9)$
$N_7 = k_{11}k_{13}k_{14}k_{16}(k_2+k_3)k_7(k_5+k_8)$
$N_8 = k_{11}k_{13}(k_{15}+k_{16})(k_2+k_3)k_4k_7k_8$
$N_9 = k_{10}k_{13}(k_{15}+k_{16})(k_2+k_3)k_5k_6k_9$
$N_{11} = k_{13}k_{14}k_{16}(k_2+k_3)k_5(k_{10}+k_7)k_9$
$D_1 = k_1(k_{15}+k_{16})k_3(k_{10}(k_{12}(k_5+k_8)+k_5k_9)+$
$k_7(k_{11}(k_5+k_8)+k_{12}(k_5+k_8)+k_5k_9))$
$D_2 = k_{12}(k_{15}+k_{16})(k_2+k_3)k_4(k_{10}k_7)k_8$
$D_3 = k_{10}k_{12}(k_{15}+k_{16})(k_2k_3)k_6(k_5+k_8)$
$D_4 = k_{13}(k_{15}+k_{16})(k_2+k_3)(k_{11}k_7(k_5+k_8)+k_5(k_{10}+k_7)k_9)$
$D_5 = k_{14}k_{16}(k_2+k_3)(k_{10}(k_{12}(k_5+k_8)+k_5k_9)+k_7(k_{11}(k_5+k_8)+$
$k_{12}(k_5+k_8)+k_5k_9))$
$D_6 = k_1(k_{15}+k_{16})k_4(k_{11}k_3(k_7k_8)+k_{10}(k_{12}(k_3+k_8)+k_3(k_8+k_9))+$
$k_7(k_{12}(k_3+k_8)+k_3(k_8+k_9)))$ (S21-S42)
$D_7 = k_1(k_{15}+k_{16})k_6(k_3(k_{12}k_5+k_{12}k_8+k_{11}(k_5+k_8)+k_5k_9)+$
$k_{10}(k_{12}(k_5+k_8)+k_3(k_5+k_8+k_9)))$
$D_8 = k_1k_{14}(k_{16}+k_3)(k_{10}(k_{12}(k_5+k_8)+k_5k_9)+k_7(k_{11}(k_5+k_8)+$
$k_{12}(k_5+k_8)+k_5k_9))$
$D_9 = k_{13}(k_{15}+k_{16})(k_2+k_3)k_4(k_{11}k_3(k_7k_8)+(k_{10}+k_7)(k_8+k_9))$
$D_{10} = k_{13}(k_{15}+k_{16})(k_2+k_3)k_6(k_{11}(k_5+k_8)+k_5k_9+k_{10}(k_5+k_8+k_9))$
$D_{11} = k_{13}k_{14}(k_2+k_3)(k_{11}(k_{16}+k_7)(k_5+k_8)+k_{10}(k_5k_9+k_{16}(k_5+k_8+k_9))+$
$k_7(k_5k_9+k_{16}(k_5+k_8+k_9)))$

Symbols: A, 1,3-BPG; B, 3PG; C, 2PG; D,2,3-BPG; H, 2PG or Pi

Parameter	Value
e_t (M)	4.1E–07
k_2 (s^{-1})	400
k_3 (s^{-1})	9.9
k_4 (M^{-1} s^{-1})	1.85E+08
k_5 (M^{-1} s^{-1})	1.00E+08
k_6 (s^{-1})	1000
k_7 (s^{-1})	1000
k_8 (s^{-1})	10000
k_9 (s^{-1})	0.55
k_{10} (s^{-1})	1979
k_{11} (s^{-1})	0.01
k_{12} (s^{-1})	1000
k_{13} (M^{-1} s^{-1})	1800000
k_{14} (M^{-1} s^{-1})	1.00E+09
k_{15} (s^{-1})	610000
k_{16} (s^{-1})	0.19

Parameter values were taken from [3].

PRPPsyn

$$v = \frac{V_m\left(\mathrm{AB} - \dfrac{\mathrm{PQ}}{K_{eq}}\right)}{K_{m,\mathrm{A}}\,\mathrm{B} + K_{m,\mathrm{B}}\,\mathrm{A} + \dfrac{K_v K_{m,\mathrm{Q}}\,\mathrm{P}}{K_{eq}} + \dfrac{K_v K_{m,\mathrm{P}}\,\mathrm{Q}}{K_{eq}} + \mathrm{AB} + K_v\,\mathrm{P}} \qquad \text{(S43)}$$

Symbols: A, R5P; B, MgATP; P, PRPP; Q, AMP

Parameter	Value
$K_{m.\mathrm{AMP}}$ (M)	2.75E–04
$K_{m.\ \mathrm{ATP}}$ (M)	1.70E–04
K_{eq}	28.6
$K_{m.\ \mathrm{PRPP}}$ (M)	9.00E–05
$K_{m.\mathrm{R5P}}$ (M)	6.50E–04
K_v (M)	7.50E–04
V_m (M h^{-1})	5.54E–04

Parameter values were taken from [4].

Supporting Information (Nishino *et al.*)
Text S1.

AK

$$v = \frac{V_m \mathrm{AB}}{K_{i,\mathrm{A}} K_{m,\mathrm{B}} + K_{m,\mathrm{A}} \mathrm{B} + K_{m,\mathrm{B}} \mathrm{A} + \mathrm{AB}} \qquad \text{(S44)}$$

Symbols: A, ADO; B, MgATP

Parameter	Value
$K_{i,\,\mathrm{ADO}}$ (M)	5.40E−07
$K_{m,\,\mathrm{ADO}}$ (M)	1.75E−06
$K_{m,\,\mathrm{MgATP}}$ (M)	2.70E−05
V_m (M s^{-1})	5.50E−07

Parameter values were taken from [5] and [6].

APK

$$v = \frac{e_t \left(k_{catf} \dfrac{\mathrm{AB}}{K_{m,\mathrm{A}} K_{m,\mathrm{B}}} - k_{catr} \dfrac{\mathrm{PQ}}{K_{m,\mathrm{P}} K_{m,\mathrm{Q}}} \right)}{1 + \dfrac{\mathrm{A}}{K_{m,\mathrm{A}}} + \dfrac{\mathrm{B}}{K_{m,\mathrm{B}}} + \dfrac{\mathrm{AB}}{K_{m,\mathrm{A}} K_{m,\mathrm{B}}} + \dfrac{\mathrm{P}}{K_{m,\mathrm{P}}} + \dfrac{\mathrm{Q}}{K_{m,\mathrm{Q}}} + \dfrac{\mathrm{PQ}}{K_{m,\mathrm{P}} K_{m,\mathrm{Q}}}} \qquad \text{(S45)}$$

Symbols: A, ADP; B, MgADP; P, AMP; Q, MgATP

Parameter	Value
e_t (M)	9.70E−07
$K_{m,\mathrm{ADP}}$ (M)	1.00E−05
$K_{m,\mathrm{MgADP}}$ (M)	1.00E−04
$K_{m,\mathrm{MgATP}}$ (M)	1.10E−04
$K_{m,\mathrm{AMP}}$ (M)	6.70E−05
k_{catf} (s^{-1})	2080
k_{catr} (s^{-1})	3800

Parameter values were taken from [2].

ATPase, AMPase, IMPase, GSHox, non-glycolytic NADH consumption

$v = k\,\mathrm{S}$ (S46)

S: substrate of the reaction

Parameter	Value
AMPase [a]	
k (h^{-1})	1.58
ATPase [c]	
k (h^{-1})	7.12E–01
IMPase [a]	
k (h^{-1})	9.00E–02
GSHox [c]	
k (s^{-1})	2.38E–05
NADHox [b]	
k (s^{-1})	1.63E–02

Parameter values were taken from [a][7], [b][2] or [c]adjusted to achieve the appropriate steady-state concentration of metabolites.

ADPRT, HGPRT

$$v = V_m \left(\frac{\mathrm{A}}{1 + K_{m,\mathrm{A}}} \right) \left(\frac{\mathrm{B}}{1 + K_{m,\mathrm{B}}} \right) \quad \text{(S47)}$$

Symbols: (ADPRT) A, ADE; B, PRPP (HGPRT) A, PRPP; B, HX

Parameter	Value
ADPRT	
$K_{m\mathrm{.ADE}}$ (M)	2.30E–06
$K_{m\mathrm{.PRPP}}$ (M)	1.95E–05
V_m (M h^{-1})	7.80E–05
HGPRT	
$K_{m\mathrm{.PRPP}}$ (M)	5.00E–06
$K_{m\mathrm{.HX}}$ (M)	2.20E–04
V_m (M h^{-1})	2.01E–04

Parameter values were taken from [7].

PRM, PNPase

$v = ka\,\mathrm{A} - kd\,\mathrm{P}$ (S48)

Symbols: (PNPase) A, INO; P, HX. (PRM) A, R1P; P, R5P.

Parameter	Value
PNPase	
ka (s^{-1})	1.11E+03
kd (s^{-1})	1.00E+02
PRM	
ka (s^{-1})	7.25
kd (s^{-1})	1.00E+02

Parameter values were taken from [8].

PNPase2 (guanosine phosphorylation process)

$v = k\mathrm{AB}$ (S49)

Symbols: A, GUO; B, Pi

Parameter	Value
k (s^{-1}•M^{-2})	1.0E+08

Parameter value was determined by fitting the GUO depletion curve as measured by CE-TOFMS experiments (Figure S1).

6PGLase, ADA, AMPDA

$$v = \frac{V_m\,\mathrm{S}}{K_m + \mathrm{S}} \quad \text{(S50)}$$

S: substrate of the reaction

Parameter	Value
6PGLase [a]	
K_m (M)	7.99E−05
V_m (M h^{-1})	2.2518
ADA [b]	
K_m (M)	5.20E−05
V_m (M h^{-1})	2.00E−02
AMPDA [b]	
K_m (M)	8.00E−04
V_m (M h^{-1})	1.00E−05

Parameter values were taken from [a][4], [b][7].

Supporting Information (Nishino *et al.*)
Text S1.

G6PDH

$$v = \frac{V_m \dfrac{\mathrm{AB}}{K_{m,\mathrm{A}} K_{m,\mathrm{B}}}}{1 + \dfrac{\mathrm{B}}{K_{m,\mathrm{B}}}\left(1 + \dfrac{\mathrm{A}}{K_{m,\mathrm{A}}}\right) + \dfrac{\mathrm{P}}{K_{m,\mathrm{P}}} + \dfrac{\mathrm{ATP}}{K_{\mathrm{ATP}}} + \dfrac{2{,}3\text{-BPG}}{K_{2,3\text{-BPG}}}} \qquad \text{(S51)}$$

Symbols: A, G6P; B, NADP; P, NAPDH

Parameter	Value
K_{ATP} (M)	7.49E−04
$K_{2.3\mathrm{BPG}}$ (M)	2.29E−03
$K_{m.\mathrm{G6P}}$ (M)	6.67E−05
$K_{m.\mathrm{NADP}}$ (M)	3.67E−06
$K_{m.\mathrm{NADPH}}$ (M)	3.12E−06
V_m (M s^{-1})	6.40E-05

Parameter values were taken from [9].

6PGODH

$$v = \frac{e_t(N_1\mathrm{AB} - N_2\mathrm{PQ})}{D_1 + D_2\mathrm{A} + D_3\mathrm{B} + D_4\mathrm{P} + D_5\mathrm{Q} + D_6\mathrm{AB} + D_7\mathrm{AP} + D_8\mathrm{BQ} + D_9\mathrm{PQ} + D_{10}\mathrm{ABP} + D_{11}\mathrm{BPQ}} \qquad \text{(S52)}$$

$$\begin{aligned}
N_1 &= k_1k_3k_5k_7k_9 \\
N_2 &= k_2k_4k_6k_8k_{10} \\
D_1 &= k_2k_9(k_4k_6 + k_5k_6 + k_5k_7) \\
D_2 &= k_1k_9(k_4k_6 + k_5k_6 + k_5k_7) \\
D_3 &= k_3k_5k_7k_9 \\
D_4 &= k_2k_4k_6k_8 \\
D_5 &= k_2k_{10}(k_4k_6 + k_5k_6 + k_5k_7) \\
D_6 &= k_1k_3(k_5k_7 + k_5k_9 + k_6k_9 + k_7k_9) \\
D_7 &= k_1k_4k_6k_8 \\
D_8 &= k_3k_5k_7k_{10} \\
D_9 &= k_8k_{10}(k_2k_4 + k_2k_5 + k_2k_6 + k_4k_6) \\
D_{10} &= k_1k_3k_8(k_5 + k_6) \\
D_{11} &= k_3k_8k_{10}(k_5 + k_6)
\end{aligned} \qquad \text{(S53-S65)}$$

Symbols: A, NADP; B, GO6P; P, RU5P; Q, NADPH

Supporting Information (Nishino *et al.*)
Text S1.

Parameter	Value
e_t (M)	2.10E–06
k_1 (M^{-1} s^{-1})	2.40E+06
k_2 (s^{-1})	4.10E+02
k_3 (M^{-1} s^{-1})	2.00E+09
k_4 (M^{-1} s^{-1})	2.60E+04
k_5 (s^{-1})	48.0
k_6 (s^{-1})	30.0
k_7 (s^{-1})	6.30E+02
k_8 (M^{-1} s^{-1})	3.60E+04
k_9 (s^{-1})	8.00E+02
k_{10} (M^{-1} s^{-1})	2.25E+05
k_{11} (s^{-1})	3.00E+02
k_{12} (M^{-1} s^{-1})	4.95E+06

Parameter values were taken from [4].

GSSGR

$$v = \frac{e_t(N_1\mathrm{AB} - N_2\mathrm{P}^2\mathrm{Q})}{GSSGRrd} \qquad \text{(S66)}$$

$$\begin{aligned} GSSGR_{rd} &= D_1 + D_2\mathrm{A} + D_3\mathrm{B} + D_4\mathrm{P} + D_5\mathrm{Q} + D_6\mathrm{AB} + D_7\mathrm{AP} \\ &+ D_8\mathrm{BQ} + D_9\mathrm{P}^2 + (D_{10} + D_{11})\mathrm{PQ} + (D_{12} + D_{13})\mathrm{ABP} + D_{14}\mathrm{AP}^2 \\ &+ D_{15}\mathrm{BPQ} + D_{16}\mathrm{P}^2\mathrm{Q} + D_{17}\mathrm{ABP}^2 + D_{18}\mathrm{BP}^2\mathrm{Q} \end{aligned} \qquad \text{(S67)}$$

$$\begin{aligned}
N_1 &= k_1k_3k_5k_7k_9k_{11} \\
N_2 &= k_2k_4k_6k_8k_{10}k_{12} \\
D_1 &= k_2k_9k_{11}(k_4k_6 + k_4k_7 + k_5k_7) \\
D_2 &= k_1k_9k_{11}(k_4k_6 + k_4k_7 + k_5k_7) \\
D_3 &= k_3k_5k_7k_9k_{11} \\
D_4 &= k_2k_4k_6k_8k_{11} \\
D_5 &= k_2k_9k_{12}(k_4k_6 + k_4k_7 + k_5k_7) \\
D_6 &= k_1k_3(k_5k_9k_{11} + k_6k_9k_{11} + k_7k_9k_{11} + k_5k_7k_9 + k_5k_7k_{11}) \\
D_7 &= k_1k_4k_6k_8k_{11} \\
D_8 &= k_3k_5k_7k_9k_{12} \\
D_9 &= k_2k_4k_6k_8k_{10} \\
D_{10} &= k_2k_4k_6k_8k_{12} \\
D_{11} &= k_2k_{10}k_{12}(k_4k_6 + k_4k_7 + k_5k_7) \\
D_{12} &= k_1k_3k_8k_{11}(k_5 + k_6) \\
D_{13} &= k_1k_3k_5k_7k_9k_{10} \\
D_{14} &= k_1k_4k_6k_8k_{10} \\
D_{15} &= k_3k_5k_7k_{10}k_{12} \\
D_{16} &= k_8k_{10}k_{12}(k_2k_4 + k_2k_5 + k_2k_6 + k_4k_6) \\
D_{17} &= k_1k_3k_8k_{10}(k_5 + k_6) \\
D_{18} &= k_3k_8k_{10}k_{12}(k_5 + k_6)
\end{aligned} \qquad \text{(S68-S87)}$$

Symbols: A, NADPH; B, GSSG;P, GSH; Q, NADP

Parameter	Value
e_t (M)	1.25E–07
k_1 (M^{-1} s^{-1})	8.50E+07
k_2 (s^{-1})	5.10E+02
k_3 (M^{-1} s^{-1})	1.00E+08
k_4 (M^{-1} s^{-1})	5.60E+05
k_5 (s^{-1})	8.10E+02
k_6 (s^{-1})	1.00E+03
k_7 (s^{-1})	1.00E+06
k_8 (M^{-1} s^{-1})	5.00E+07
k_9 (s^{-1})	1.00E+06
k_{10} (M^{-1} s^{-1})	5.00E+07
k_{11} (s^{-1})	7.00E+03
k_{12} (M^{-1} s^{-1})	1.00E+08

Parameter values were taken from [4].

R5PI, X5PI

$$v = \frac{e_t\left(\dfrac{k_3\mathrm{A}}{K_{m,\mathrm{A}}} - \dfrac{k_2\mathrm{P}}{K_{m,\mathrm{P}}}\right)}{1 + \dfrac{\mathrm{A}}{K_{m,\mathrm{A}}} + \dfrac{\mathrm{P}}{K_{m,\mathrm{P}}}} \qquad \text{(S88)}$$

$$K_{m,\mathrm{A}} = \frac{k_2 + k_3}{k_1}, K_{m,\mathrm{P}} = \frac{k_2 + k_3}{k_4} \qquad \text{(S89)}$$

Symbols: (R5PI) A, RU5P; P, R5P. (X5PI) A, RU5P; P, X5P.

Parameter	Value
R5PI	
e_t (M)	1.42E–05
k_1 (M^{-1} s^{-1})	6.09E+04
k_2 (s^{-1})	33.3
k_3 (s^{-1})	14.2
k_4 (M^{-1} s^{-1})	2.16E+04
X5PI	
e_t (M)	4.22E–06
k_1 (M^{-1} s^{-1})	3.91E+06
k_2 (s^{-1})	4.38E+02
k_3 (s^{-1})	3.05E+02
k_4 (M^{-1} s^{-1})	1.49E+06

Parameter values were taken from [4].

TA, TK1, TK2

$$v = \frac{e_t(N_1\mathrm{AB} - N_2\mathrm{PQ})}{D_1\mathrm{A} + D_2\mathrm{B} + D_3\mathrm{P} + D_4\mathrm{Q} + D_5\mathrm{AB} + D_6\mathrm{PQ} + D_7\mathrm{BQ} + D_8\mathrm{AP}} \quad \text{(S90)}$$

$$\begin{aligned}
N_1 &= k_1k_3k_5k_7 \\
N_2 &= k_2k_4k_6k_8 \\
D_1 &= k_1k_3(k_6 + k_7) \\
D_2 &= k_5k_7(k_2 + k_3) \\
D_3 &= k_2k_4(k_6 + k_7) \\
D_4 &= k_6k_8(k_2 + k_3) \\
D_5 &= k_1k_5(k_3 + k_7) \\
D_6 &= k_4k_8(k_2 + k_6) \\
D_7 &= k_5k_8(k_2 + k_3) \\
D_8 &= k_1k_4(k_6 + k_7)
\end{aligned} \quad \text{(S91-S100)}$$

Symbols: (TA) A, S7P; B, GA3P; P, E4P; Q, F6P (TK1) A, X5P; B, R5P; P, GA3P; Q, S7P (TK2) A, X5P; B, E4P; P, GA3P; Q, F6P

Supporting Information (Nishino *et al.*)
Text S1.

Parameter	Value
TA	
e_t (M)	6.90E–07
k_1 (M^{-1} s^{-1})	2.16E+04
k_2 (s^{-1})	45.3
k_3 (s^{-1})	16.3
k_4 (M^{-1} s^{-1})	3.00E+04
k_5 (M^{-1} s^{-1})	4.90E+05
k_6 (s^{-1})	60.0
k_7 (s^{-1})	17.0
k_8 (M^{-1} s^{-1})	7.90E+04
TK1	
e_t (M)	3.30E–07
k_1 (M^{-1} s^{-1})	2.16E+05
k_2 (s^{-1})	38.0
k_3 (s^{-1})	34.0
k_4 (M^{-1} s^{-1})	1.56E+05
k_5 (M^{-1} s^{-1})	3.29E+05
k_6 (s^{-1})	1.75E+02
k_7 (s^{-1})	40.0
k_8 (M^{-1} s^{-1})	4.48E+04
TK2	
e_t (M)	3.30E–07
k_1 (M^{-1} s^{-1})	2.16E+05
k_2 (s^{-1})	38.0
k_3 (s^{-1})	34.0
k_4 (M^{-1} s^{-1})	1.56 E+05
k_5 (M^{-1} s^{-1})	2.24E+06
k_6 (s^{-1})	1.75E+02
k_7 (s^{-1})	40.0
k_8 (M^{-1} s^{-1})	2.13E+04

Parameter values were taken from [4].

Supporting Information (Nishino *et al.*)
Text S1.

L_GCS

$$v=\frac{\dfrac{V_m\,\mathrm{ABC}}{\alpha K_{m,\mathrm{A}}K_{m,\mathrm{Glu}}^{app}K_{m,\mathrm{C}}}}{1+\dfrac{\mathrm{B}}{K_{m,\mathrm{Glu}}^{app}}+\dfrac{\mathrm{BC}}{K_{m,\mathrm{Glu}}^{app}K_{m,\mathrm{C}}}+\dfrac{\mathrm{AB}}{K_{m,\mathrm{A}}K_{m,\mathrm{Glu}}^{app}}+\dfrac{\mathrm{ABC}}{\alpha K_{m,\mathrm{A}}K_{m,\mathrm{Glu}}^{app}K_{m,\mathrm{C}}}} \qquad \text{(S101)}$$

$$K_{m,\mathrm{Glu}}^{app}=\frac{K_{m,\mathrm{Glu}}(1+\mathrm{GSH})}{K_{i,\mathrm{GSH}}} \qquad \text{(S102)}$$

Symbols: A, MgATP; B, glutamate; C, cysteine

Parameter	Value
$K_{i,\mathrm{GSH}}$ (M)	3.40E−03
$K_{m,\mathrm{MgATP}}$ (M)	4.00E−04
$K_{m,\mathrm{Glu}}$ (M)	1.80E−03
$K_{m,\mathrm{C}}$ (M)	1.00E−04
V_m (M h^{-1})	5.00E−02
α	0.20

Parameter values were taken from [10].

GSH_S

$$v=\frac{\dfrac{V_m\,\mathrm{ABC}}{\alpha K_{m,\mathrm{A}}K_{m,\mathrm{B}}K_{m,\mathrm{C}}}}{1+\dfrac{\mathrm{A}}{K_{m,\mathrm{A}}}+\dfrac{\mathrm{AB}}{K_{m,\mathrm{A}}K_{m,\mathrm{B}}}+\dfrac{\mathrm{AC}}{K_{m,\mathrm{A}}K_{m,\mathrm{C}}}+\dfrac{\mathrm{ABC}}{\alpha K_{m,\mathrm{A}}K_{m,\mathrm{B}}K_{m,\mathrm{C}}}} \qquad \text{(S103)}$$

Symbols: A, L_GC; B, glycine; C, MgATP

Parameter	Value
$K_{m,\mathrm{L\_GC}}$ (M)	9.90E−03
$K_{m,\mathrm{glycine}}$ (M)	1.37E−03
$K_{m,\mathrm{MgATP}}$ (M)	2.30E−03
V_m (M h^{-1})	8.84E−02
α	2.60

Parameter values were taken from [4].

Supporting Information (Nishino *et al.*)
Text S1.

GSSGtransport

$$v = V_m \left(\frac{\mathrm{GSSG}}{\mathrm{GSSG} + K_{m,\mathrm{GSSG}}} \right) \left(\frac{\mathrm{MgATP}}{\mathrm{MgATP} + K_{m,\mathrm{MgATP}}} \right) \quad \text{(S104)}$$

Parameter	Value
$K_{m,\mathrm{GSSG}}$ (M)	1.00E−04
$K_{m,\mathrm{MgATP}}$ (M)	6.30E−04
V_m (M h^{-1})	1.90E−04

Parameter values were taken from [4].

HXtr

$$v = P_m \mathrm{HX}_{in} + \frac{V_m \mathrm{HX}_{in}}{\mathrm{HX}_{in} + K_m} - \frac{V_{min} \mathrm{HX}_{ex}}{K_{min}\left(1 + \frac{\mathrm{ADE}_{ex}}{K_i}\right) + \mathrm{HX}_{ex}} \quad \text{(S105)}$$

Parameter	Value
K_i (M)[a]	1.20E−05
K_m (M)	4.00E−04
K_{min} (M)	1.80E−04
P_m (h^{-1})	37.8
V_m (M h^{-1})	0.1516
V_{min} (M h^{-1})	0.1008

Parameter values were taken from [7] and [11].

LACtr, PYRtr, Pitr

$$v = k_0 \mathrm{X}_{in} - k_1 \mathrm{X}_{ex} \quad \text{where} \quad k_0 = k_1 / K_{eq} \quad \text{(S106)}$$

$$K_{eq} = 1 + \frac{10^{pHi - pKa}}{1 + 10^{pHi - pKa} r^{-1}} \quad \text{(S107)}$$

Calculated using Donnan ratio (r) = 0.69, *pKa* (PYR) = 2.39, *pKa* (LAC) = 0.00506, *pKa* (Pi) = 6.75 where Hct = 0.5.
X: LAC, PYR, Pi

Parameter	Value
LACtr	
k_0 (s^{-1})	7.33E–03
k_1 (s^{-1})	5.06E–03
PYRtr	
k_0 (s^{-1})	2.61E–02
k_1 (s^{-1})	1.80E–02
Pitr	
k_0 (s^{-1})	6.06E–04
k_1 (s^{-1})	5.60E–04

Parameter values were taken from [2].

INOtr, ADOtr, ADE tr

$$v = V_m\left(\frac{X_{in}}{K_m + X_{in}} - \frac{X_{ex}}{K_m + X_{ex}}\right) \qquad \text{(S108)}$$

X: INO, ADO, ADE

Parameter	Value
ADEtr	
K_m (M)	2.60E–03
V_m (M h^{-1})	90.0E–02
ADOtr	
K_m (M)	1.20E–04
V_m (M h^{-1})	6.12E–02
INOtr	
K_m (M)	1.20E–04
V_m (M h^{-1})	6.12E–02

Parameter values were taken from [7].

Supporting Information (Nishino *et al.*)
Text S1.

Na⁺/K⁺ pump

$$v = \frac{\left(\frac{\mathrm{ATP}}{\mathrm{ATP}+K_m}\right)\left(\frac{V_m}{2}\right)\left((\mathrm{K}^+{}_{ex})^2 + \frac{B_2\,\mathrm{K}^+{}_{ex}z}{2}\right)}{Pump_{rd}} \qquad \text{(S109)}$$

$$Pump_{rd} = B_1B_2 + 2B_2\,\mathrm{K}^+{}_{ex} + (\mathrm{K}^+{}_{ex})^2 + \left(\frac{B_3}{\mathrm{Na}^+{}_{in}} + 1\right)^3 \left(B_1B_2k_2k_1 + k_3k_1\,(\mathrm{K}^+{}_{ex})^2 + B_2\,\mathrm{K}^+{}_{ex}z\right) \qquad \text{(S110)}$$

Parameter	Value
B_1 (M)	6.17E−05
B_2 (M)	1.33E−04
B_3 (M)	6.27E−03
K_m (M)	7.64E−04
V_m (M h^{-1})	2.32E−03
$k_2\,k_1$	8.20E−03
$k_3\,k_1$	5.01E−02
z	0.711

Parameter values were taken from [7].

Na⁺, K⁺ leak

$$v = \frac{K_x\,z\log(r)\left(\mathrm{X}_{ex} - r\,\mathrm{X}_{in}\right)}{r-1} + \frac{V_m\mathrm{X}_{ex}}{\left(K_m + \mathrm{X}_{ex}\right) - r\frac{\mathrm{X}_{in}}{\left(K_m + r\,\mathrm{X}_{in}\right)}} \qquad \text{(S111)}$$

X : Na^+, K^+

Parameter	Value
K^+Leak	
K_m (M)	4.00E−03
K_x (M)	6.35E−06
V_m (M h^{-1})	3.12E−03
r	0.620
z	1.00
Na^+Leak	
K_m (M)	2.10E−02
K_x (M)	7.06E−06
V_m (M h^{-1})	2.82E−03
r	0.620
z	1.00

Parameter values were taken from [7].

1-2. Intracellular pH degradation profile of PAGGGM-stored RBCs

The intracellular pH decline in PAGGGM-stored RBC was approximated as eq.(S112) from the estimated pH time-series under 4°C (Figure 2A).

$$\mathrm{pH(t)} = 8.79 \times 10^{-14} t^2 - 6.01 \times 10^{-7} t + 7.62 \qquad \text{(S112)}$$

1-3. Descriptions of binding processes of metabolites to Mg^{2+}, oxyHb and deoxyHb.

The binding processes between metabolites to Mg^{2+} and hemoglobin are also considered in the model. Kinetics and association constants for calculation of these bindings were shown below:

Binding of metabolites to hemoglobin

Binding substrates		Complex
deoxyHb +	↔	deoxyHb1,3-BPG
deoxyHb +	↔	deoxyHb2,3-BPG
deoxyHb + fADP	↔	deoxyHbADP
deoxyHb + fATP	↔	deoxyHbATP
deoxyHb +	↔	deoxyHbF-1,6BP
deoxyHb + GDP	↔	deoxyHbGDP
deoxyHb +	↔	deoxyHbMgATP
oxyHb + 1,3-BPG	↔	oxyHb1,3-BPG
oxyHb + 2,3-BPG	↔	oxyHb2,3-BPG
oxyHb + ADP	↔	oxyHbADP
oxyHb + ATP	↔	oxyHbATP
oxyHb + MgATP	↔	oxyHbMgATP

$$v = K'_a \mathrm{AB} - K_d \mathrm{P} \qquad \text{(S113)}$$

$$K'_a = \frac{1 + \dfrac{2n}{10^{-7.2}} + \left(\dfrac{n}{10^{-7.2}}\right)^2}{1 + \dfrac{2n}{10^{-\mathrm{pH}}} + \left(\dfrac{n}{10^{-\mathrm{pH}}}\right)^2} \times K_a \qquad \text{(S114)}$$

Symbols: A and B, binding substrates; P, complex

Supporting Information (Nishino *et al.*)
Text S1.

Parameter	Value
deoxyHb1,3-BPG	
K_a	1860000
K_d	1200
N	1.00E−06
deoxyHb1,3-BPG	
K_a	6000000
K_d	1200
N	1.00E−06
deoxyHbADP	
K_a	1440000
K_d	1200
N	1.00E−06
deoxyHbATP	
K_a	3120000
K_d	1200
N	1.00E−06
deoxyHbF-1,6BP	
K_a	1212000
K_d	1200
N	1.00E−06
deoxyHbGDP	
K_a	1212000
K_d	1200
N	1.00E−06
deoxyHbMgATP	
K_a	168000
K_d	1200
N	1.00E−06
oxyHb1,3-BPG	
K_a	380000
K_d	1200
N	1.00E−06
oxyHb2,3-BPG	
K_a	300000
K_d	1200
N	1.00E−06
oxyHbADP	
K_a	300000
K_d	1200
N	1.00E−06
oxyHbATP	
K_a	432000

K_d	1200
N	1.00E–06
oxyHbMgATP	
K_a	46800
K_d	1200
N	1.00E–06

Parameter values were taken from [12].

Binding of metabolites to Mg^{2+}

Mg1,3-BPG, Mg2,3-BPG

Binding substrates		Complex
Mg^{2+} + 1,3-BPG	↔	Mg1,3-BPG
Mg^{2+} + 2,3-BPG	↔	Mg2,3-BPG

$$v = K'_a \mathrm{AB} - K_d \mathrm{P} \qquad \text{(S115)}$$

$$K'_a = \frac{0.0032K_a\left(Kmgbpg + 10^{-\mathrm{pH}} \times Khbpg \times Kmghbpg\right)}{1+\left(10^{-\mathrm{pH}} \times Khbpg\right)+\left(10^{-2\mathrm{pH}} \times Khbpg \times Kh2bpg\right)+\left(\mathrm{K}^+ \times Khbpg\right)+\left(\mathrm{K}^+ \times 10^{-\mathrm{pH}} \times Khbpg \times Kkhbpg\right)} \qquad \text{(S116)}$$

Symbols: A and B, binding substrates; P, complex

Parameter	Value
Mg1,3-BPG	
K_a	228000
K_d	1200
Kh2bpg	4270000
Khbpg	162000000
Kkbpg	85.1
Kkhbpg	8.9
Kmgbpg	7410
Kmghbpg	513
Mg2,3-BPG	
K_a	804000
K_d	1200
Kh2bpg	4270000
Khbpg	162000000
Kkbpg	85.1
Kkhbpg	8.9
Kmgbpg	7410
Kmghbpg	513

Parameter values were taken from [12].

Supporting Information (Nishino *et al.*)
Text S1.

MgAMP

Binding substrates		Complex
Mg^{2+} + AMP	$\leftrightarrow$	MgAMP

$v = K_a \mathrm{AB} - K_d \mathrm{P}$ (S117)

Symbols: A and B, binding substrates; P, complex

Parameter	Value
K_a	54054
K_d	1200

Parameter values were taken from [12].

MgADP

Binding substrates		Complex
Mg^{2+} + ADP	$\leftrightarrow$	MgADP

$v = K'_a \mathrm{AB} - K_d \mathrm{P}$ (S118)

$$K'_a = \frac{Kmgadp + \left(10^{-\mathrm{pH}} \times Khadp \times Kmgadp\right)}{1 + \left(10^{-\mathrm{pH}} \times Khadp\right) + \left(\mathrm{K}^{+} \times Kkadp\right)} \times K_a \qquad \text{(S119)}$$

Symbols: A and B, binding substrates; P, complex

Parameter	Value
K_a	1711.2
K_d	1200
Khadp	5420000
Kkadp	4.8
Kmgadp	3290
Kmghadp	107

Parameter values were taken from [12].

Supporting Information (Nishino *et al.*)
Text S1.

MgATP

Binding substrates		Complex
Mg^{2+} + ATP	$\leftrightarrow$	MgATP

$$v = K_a' \mathrm{AB} - K_d \mathrm{P} \qquad \text{(S120)}$$

$$K_a' = \frac{Kmgadp + \left(10^{-\mathrm{pH}} \times Khadp \times Kmgadp\right)}{1 + \left(10^{-\mathrm{pH}} \times Khadp\right) + \left(\mathrm{K}^+ \times Kkadp\right)} \times K_a \qquad \text{(S121)}$$

Symbols: A and B, binding substrates; P, complex

Parameter	Value
K_a	2620.8
K_d	1200
Khatp	9070000
Kkatp	14.0
Kmgatp	43200
Kmghatp	748.0

Parameter values were taken from [12].

MgF-1,6BP, MgGDP

Binding substrates		Complex
Mg^{2+} + F-1,6-BPG	$\leftrightarrow$	MgF-1,6BP
Mg^{2+} + GDP	$\leftrightarrow$	MgGDP

$$v = K_{a,A}' \mathrm{AB} - K_{d,A} \mathrm{P} \qquad \text{(S122)}$$

$$K_{a,A}' = \frac{0.0083 K_{a,A}\left(Kmg,A + 10^{-\mathrm{pH}} \times Kh,A \times Kmgh,A\right)}{1 + \left(10^{-\mathrm{pH}} \times Kh,A\right) + \left(10^{-2\mathrm{pH}} \times Kh,A \times Kh2,A\right) + \left(\mathrm{K}^+ \times Kh,A\right) + \left(\mathrm{K}^+ \times 10^{-\mathrm{pH}} \times Kh,A \times Kkh,A\right)}$$

(S123)

A, B, binding substrates; P, complex, *A*, *f16bpg* or *gdp.*

Supporting Information (Nishino *et al.*)
Text S1.

Parameter	Value
MgF-1,6-BP	
$K_{a,f16bpg}$	480000
$K_{d,f16bpg}$	1200
Kh2,f16bpg	1120000
Kh,f16bpg	7560000
Kk,f16bpg	10.7
Kkh,f16bpg	3.3
Kmg,f16bpg	363.0
Kmgh,f16bpg	89.0
MgGDP	
$K_{a,gdp}$	480000
$K_{d,gdp}$	1200
Kh2,gdp	1120000
Kh,gdp	7560000
Kk,gdp	10.7
Kkh,gdp	3.3
Kmg,gdp	363.0
Kmgh,dgp	89.0

Parameter values were taken from [12].

MgPi

Binding substrates		Complex
Mg^{2+} + Pi	↔	MgPi

$$v = K'_a \mathrm{AB} - K_d \mathrm{P} \qquad \text{(S124)}$$

$$K'_a = \frac{10^{-7.2} \times Khpi + 0.15Kkpi}{1 + \left(10^{-\mathrm{pH}} \times Khpi\right) + \left(\mathrm{K}^+ \times Kkpi\right)} \times K_a \qquad \text{(S125)}$$

A, B, binding substrates; P, complex

Parameter	Value
K_a	40800
K_d	1200
Khpi	5680000
Kkpi	3.0

Parameter values were taken from [12].

1-4. Descriptions of Band3 protein mediated interactions between hemoglobin and glycolytic enzymes.

Reversible binding of some glycolytic enzymes and two allosteric form of hemoglobin to Band3 protein, Kinetics and association constants for calculation of these bindings were shown below:

Binding proteins		Complex
Band3 + ALD	↔	Band3-ALD
Band3 + GAPDH	↔	Band3-GAPDH
Band3 + PFK	↔	Band3-PFK
Band3 + deoxyHb	↔	Band3-deoxyHb
Band3 + oxyHb	↔	Band3-oxyHb

$$v = K_a \mathrm{AB} - K_d \mathrm{P} \qquad \text{(S126)}$$

Symbols: A,B, binding proteins; P, complex

Parameter	Value
Band3-ALD[a]	
K_a	1200000000
K_d	1200
Band3-GAPDH[a]	
K_a	2400000000
K_d	1200
Band3-PFK[b]	
K_a	6000000000
K_d	1200
Band3-deoxyHb[c]	
K_a	12000000
K_d	1200
Band3-oxyHb[c]	
K_a	120000
K_d	1200

Parameter values were taken from [a][13], [b][14], [c][15].

1-5. Initial and steady-state concentrations of all substrates in the model

Abbreviation of metabolites and enzymes are corresponding to those shown in Table 1 in the main text.

Substrate	Concentration(M)
ADE	1.53E–05
ADO	4.62E–08
DHAP	1.51E–05
E4P	4.57E–07
F6P	1.94E–05
F1,6-BP	5.35E–06
G6P	5.96E–05
GA3P	3.69E–06
GDP	8.77E–05
GL6P	5.33E–09
GLC	4.75E–02
GO6P	4.47E–05
GSH	3.27E–03
GSSG	4.65E–06
HX	1.61E–06
IMP	8.06E–06
INO	1.45E–07
LAC	1.33E–03
L_GC	4.22E–07
NAD	6.51E–05
NADH	2.40E–07
NADP	6.47E–08
NADPH	6.53E–05
Na^+	3.39E–02
K^+	1.33E–01
PEP	8.19E–06
PRPP	1.41E–06
PYR	5.16E–05
Pi	1.01E–03
R1P	8.08E–05
R5P	5.86E–06
RU5P	4.93E–06
S7P	2.10E–05
X5P	8.99E–06
1,3-BPG	2.15E–07
2PG	1.45E–05
3PG	4.77E–05

Continued from the preceding page

2,3-BPG (free)	8.09E–04
2,3-BPG (total)	3.68E–03
AMP	2.48E–05
ADP	6.28E–05
ATP (free)	5.51E–05
ATP (total)	1.91E–03
glutamate	2.00E–04
glycine	1.80E–04
cycteine	2.00E–07
ADE (extracellular)	1.44E–03
GUO (extracellular)	1.44E–03
ADO (extracellular)	0.00
HX (extracellular)	0.00
INO (extracellular)	0.00
K^+ (extracellular)	0.00
Na^+ (extracellular)	6.40E–02
LAC (extracellular)	0.00
PYR (extracellular)	0.00
Pi (extracellular)	1.60E–02
Band3 binding region (free)	2.71E–06
Band3-ALD complex	2.70E–07
Band3-GAPDH complex	6.47E–06
Band3-PFK complex	1.02E–07
ALD (free)	9.95E–05
GAPDH (free)	1.19E–03
PFK (free)	7.54E–06
Band3-deoxyHb complex	1.05E–06
Band3-deoxyHb1,3-BPG complex	3.83E–10
Band3-deoxyHb2,3-BPG complex	6.81E–06
Band3-deoxyHbADP complex	1.13E–07
Band3-deoxyHbATP complex	2.30E–07
Band3-deoxyHbF1,6-BP complex	6.07E–09
Band3-deoxyHbGDP complex	9.95E–08
Band3-deoxyHbMgATP complex	2.07E–07
Band3-oxyHb	1.30E–06
Band3-oxyHb1,3-BPG complex	9.67E–11
Band3-oxyHb2,3-BPG complex	4.21E–07
Band3-oxyHbADP complex	2.93E–08
Band3-oxyHbATP complex	3.94E–08
Band3-oxyHbF1,6-BP complex	0.00
Band3-oxyHbGDP complex	0.00
Band3-oxyHbMgATP complex	7.15E–08
deoxyHb (free)	3.86E–05

Continued from the preceding page

deoxyHb-1,3-BPG complex	1.41E–08
deoxyHb-2,3-BPG complex	2.51E–04
deoxyHb-ADP complex	4.18E–06
deoxyHb-ATP complex	8.48E–06
deoxyHb-F1,6-BP complex	2.24E–06
deoxyHb-GDP complex	3.67E–07
deoxyHb-MgATP complex	7.65E–06
oxyHb (free)	4.78E–03
oxyHb-1,3-BPG complex	3.57E–07
oxyHb-1,3-BPG complex	1.55E–03
oxyHb-ADP complex	1.08E–04
oxyHb-ATP complex	1.45E–04
oxyHb-MgATP complex	2.64E–04
Mg^{2+} (free)	6.03E–04
Mg^{2+}-1,3-BPG complex	2.92E–08
Mg^{2+}-2,3-BPG complex	5.69E–04
Mg^{2+}-ADP complex	1.30E–04
Mg^{2+}-AMP complex	9.11E–07
Mg^{2+}-ATP complex	1.41E–03
Mg^{2+}-F1,6-BP complex	1.48E–06
Mg^{2+}-GDP complex	2.43E–05
Mg^{2+}-Pi complex	2.14E–05

2. Parameter settings

In terms of the reaction activities of 3 groups (Na^+/K^+ pump activity, purine salvage activities, and all other reaction activities), we used the same parameter values which were estimated by using real-number genetic algorithm in our published paper [16], because those parameters are assumed to be altered only by cold temperature.
In GA analysis, predicted ATP and 2,3-BPG were fit to 8 points time-series (every 7days from 0 to 49 days of storage) of ATP and 2,3-BPG in cold-stored RBCs preserved in commercially available Mannitol-Adenine-Phosphate (MAP) solution [17]. The upper and lower bounds of the three adjustable enzymatic activities were 100% and 0.1% of the original activities, respectively. The population size of each generation was 300 and parameter selection was terminated at 4,000 generations. The evaluation function by using GA analysis as follows;

$$\mathrm{EV} = \frac{1}{n}\sum_{i=1}^{n}\left(\frac{[\mathrm{ATP_{exp}}]_i - [\mathrm{ATP_{pred}}]_i}{[\mathrm{ATP_{pred}}]_i}\right)^2 + \frac{1}{n}\sum_{i=1}^{n}\left(\frac{[\text{2,3-BPG}_{\mathrm{exp}}]_i - [\text{2,3-BPG}_{\mathrm{pred}}]_i}{[\text{2,3-BPG}_{\mathrm{pred}}]_i}\right)^2 \qquad \text{(S127)}$$

where EV is the error rate between the predicted and the training data, n is the number of data points (n = 8), ATP_{exp} and 2,3-BPG_{exp} represent the reference data [17], and ATP_{pred} and 2,3-BPG_{pred} are the predicted values by the model. Each parameter was normalized by its initial concentration at day 0. The reaction activities of 3 groups were estimated as 0.1%, 25.0%, and 3.0% of the values in the basal model (37°C), respectively. These parameters were searched within their feasible ranges which we provided in previous study in [16].

Rate constant of guanosine phosphorylation (k = 1.0e+8 $s^{-1}\cdot M^{-2}$) was obtained from the manually fitting to the time-series of guanosine measured by CE-TOFMS. The guanosine consumption curve calculated by the model showed good fits to the CE-TOFMS data (Figure S1). Moreover, we performed a simulation analysis to observe an influence of the rate constant of guanosine phosphorylation (k) on the dynamic behaviors of metabolic intermediates (Figure S4). In the analysis, k was varied from 1e+6 to 1e+9 and other conditions were same as PAGGGM-stored RBC model. The dynamic behaviors of some metabolic indicators, ATP and HX, were not sensitive to the guanosine phosphorylation rate. The temporal accumulation of 2,3-BPG and other metabolic intermediates were altered when the rate constant was set in lower value, but not sensitive around the value of 1e+8. Therefore, we consider that the rate constant

used in our model can be used to predict the effect of guanosine supply, as well as the effect of the ratio of adenine and guanosine on the metabolic dynamics of cold-stored RBC.

References

1. Kinoshita A, Tsukada K, Soga T, Hishiki T, Ueno Y, et al. (2007) Roles of hemoglobin Allostery in hypoxia-induced metabolic alterations in erythrocytes: simulation and its verification by metabolome analysis. J Biol Chem 282: 10731-10741.
2. Mulquiney PJ, Kuchel PW (1999) Model of 2,3-bisphosphoglycerate metabolism in the human erythrocyte based on detailed enzyme kinetic equations: equations and parameter refinement. Biochem J 342 Pt 3: 581-596.
3. Mulquiney PJ, Bubb WA, Kuchel PW (1999) Model of 2,3-bisphosphoglycerate metabolism in the human erythrocyte based on detailed enzyme kinetic equations: in vivo kinetic characterization of 2,3-bisphosphoglycerate synthase/phosphatase using 13C and 31P NMR. Biochem J 342 Pt 3: 567-580.
4. McIntyre LM, Thorburn DR, Bubb WA, Kuchel PW (1989) Comparison of computer simulations of the F-type and L-type non-oxidative hexose monophosphate shunts with 31P-NMR experimental data from human erythrocytes. Eur J Biochem 180: 399-420.
5. Hawkins CF, Bagnara AS (1987) Adenosine kinase from human erythrocytes: kinetic studies and characterization of adenosine binding sites. Biochemistry 26: 1982-1987.
6. Mimouni M, Bontemps F, Van den Berghe G (1994) Kinetic studies of rat liver adenosine kinase. Explanation of exchange reaction between adenosine and AMP. J Biol Chem 269: 17820-17825.
7. Joshi A, Palsson BO (1989) Metabolic dynamics in the human red cell. Part I--A comprehensive kinetic model. J Theor Biol 141: 515-528.
8. Messana I, Orlando M, Cassiano L, Pennacchietti L, Zuppi C, et al. (1996) Human erythrocyte metabolism is modulated by the O2-linked transition of hemoglobin. FEBS Lett 390: 25-28.
9. Buckwitz D, Jacobasch G, Kuckelkorn U, Plonka A, Gerth C (1990) Glucose-6-phosphate dehydrogenase from Plasmodium berghei: kinetic and electrophoretic characterization. Exp Parasitol 70: 264-275.
10. Nakayama Y, Kinoshita A, Tomita M (2005) Dynamic simulation of red blood cell metabolism and its application to the analysis of a pathological condition. Theor Biol Med Model 2: 18.
11. Domin BA, Mahony WB, Zimmerman TP (1988) Purine nucleobase transport in

human erythrocytes. Reinvestigation with a novel "inhibitor-stop" assay. J Biol Chem 263: 9276-9284.

12. Mulquiney PJ, Kuchel PW (2003) Modelling metabolism with Mathematica. Boca Raton, FL: CRC Press.
13. von Ruckmann B, Schubert D (2002) The complex of band 3 protein of the human erythrocyte membrane and glyceraldehyde-3-phosphate dehydrogenase: stoichiometry and competition by aldolase. Biochim Biophys Acta 1559: 43-55.
14. Jenkins JD, Madden DP, Steck TL (1984) Association of phosphofructokinase and aldolase with the membrane of the intact erythrocyte. J Biol Chem 259: 9374-9378.
15. Walder JA, Chatterjee R, Steck TL, Low PS, Musso GF, et al. (1984) The interaction of hemoglobin with the cytoplasmic domain of band 3 of the human erythrocyte membrane. J Biol Chem 259: 10238-10246.
16. Nishino T, Yachie-Kinoshita A, Hirayama A, Soga T, Suematsu M, et al. (2009) In silico modeling and metabolome analysis of long-stored erythrocytes to improve blood storage methods. J Biotechnol 144: 212-223.
17. Shiba M, Mura T, Masuyama T, Nagahashi H, Tayama T, et al. (1991) Preperation and preservation of red blood cell concentrates in MAP solution by quadruple bag system. Japanese Journal of Transfusion Medicine 37: 404-410.
